# Synthesis, Crystal Structures and Properties of Ferrocenyl Bis-Amide Derivatives Yielded via the Ugi Four-Component Reaction

**DOI:** 10.3390/molecules22050737

**Published:** 2017-05-04

**Authors:** Mei Zhao, Guang-Kui Shao, Dan-Dan Huang, Xue-Xin Lv, Dian-Shun Guo

**Affiliations:** College of Chemistry, Chemical Engineering and Materials Science, Collaborative Innovation Center of Functionalized Probes for Chemical Imaging in Universities of Shandong, Shandong Normal University, Jinan 250014, China; chmeizhao@163.com (M.Z.); shaoguangkuizhen@126.com (G.-K.S.); hdd514614@126.com (D.-D.H.); xuexinlyu@foxmail.com (X.-X.L.)

**Keywords:** ferrocenyl bis-amide, Ugi four-component reaction, synthesis, crystal structure, cyclic voltammetry

## Abstract

Ten ferrocenyl bis-amide derivatives were successfully synthesized via the Ugi four-component reaction by treating ferrocenecarboxylic acid with diverse aldehydes, amines, and isocyanides in methanol solution. Their chemical structures were fully characterized by IR, NMR, HR-MS, and X-ray diffraction analyses. They feature unique molecular morphologies and create a 14-membered ring motif in the centro-symmetric dimers generated in the solid state. Moreover, the electrochemical behavior of these ferrocenyl bis-amides was assessed by cyclic voltammetry.

## 1. Introduction

Ferrocene possesses unique structural and electronic features, and its derivatives evoke much interest in view of their widespread applications in many areas, such as medicinal chemistry [1,2], asymmetric catalysis [3,4], and materials science [5,6,7,8]. Peptides play crucial role in cellular signaling processes and account for numerous targets in biological medicine [9]. Recently, incorporating a ferrocenyl group into peptide structures has received considerable attention [10,11,12,13] as ferrocene was extensively exploited as a potential probe to identify non-covalent interactions between peptide structures, and the electron-transfer properties of different peptide secondary units. Although versatile ferrocenyl peptides or peptide-like derivatives have been reported [2,14,15,16,17,18,19], the majority of such compounds were generally obtained via a time-consuming stepwise strategy, which is usually inefficient and reduces the overall greenness of the synthesis.

Multicomponent reactions (MCRs) combine three or more reactants to yield single products in one step and offer a high degree of synthetic efficiency and atom economy. Thus, the MCRs have fast become one of the favorite strategies for the synthesis of potentially pharmacological molecules [20]. In particular, the Ugi four-component reaction (U-4CR) [21] is now a powerful tool for the construction of diverse dipeptide-like molecules in combinatorial chemistry [22]. Bazgir et al. firstly reported ferrocenyl bis/tri-amide compounds generated via the U-4CR using ferrocenealdehyde [14,15]. Very recently, utilizing the U-4CR [16], we have successfully synthesized two novel ferrocenyl bis-amide molecules from ferrocenecarboxylic acid and ferrocene-1,1′-dicarboxylic acid, respectively. In this strategy, steric ferrocene mono/di-acid was applied to facilely construct a new type of dipeptide-like ferrocenyl derivatives with excellent features.

As part of our continuous interest in developing redox-active dipeptide-like molecule libraries and evaluating the scope of this method, we further employ the U-4CR to synthesize a family of ferrocenyl bis-amide compounds by treatment of ferrocenecarboxylic acid with various aldehydes, amines, and isocyanides. Moreover, their electrochemical properties and the crystal structures of four typical compounds were presented.

## 2. Results and Discussion

### 2.1. Synthesis and Characterization

**Scheme 1:**
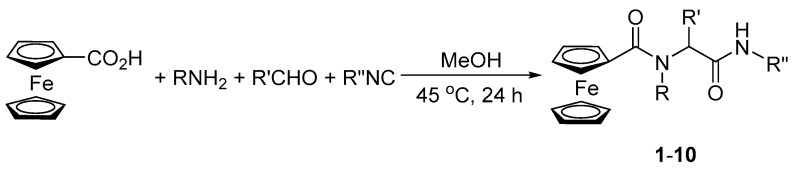
Synthetic approach to ferrocenyl bis-amides **1**–**10**.

The U-4CR is becoming the most efficient strategy for the construction of dipeptide-like compounds [14,15,16,22]. We found that ferrocenecarboxylic acid can react smoothly with a mixture of diverse amines, aldehydes, and isocyanides in the absence of any catalyst in methanol solution (Scheme 1) and afford new ferrocenyl bis-amides **1**–**10** in moderate to excellent yields. The results are summarized in Table 1.

**Scheme 2:**
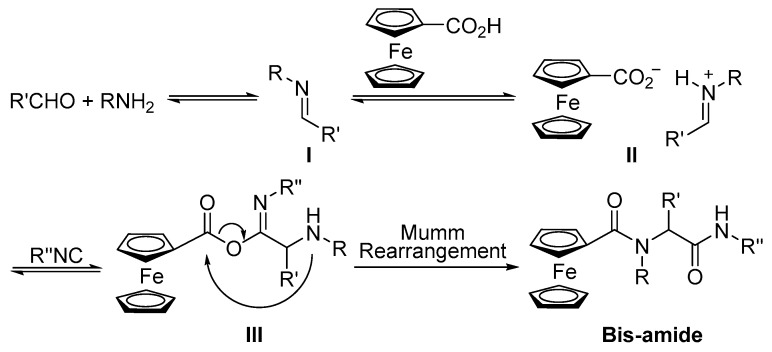
Plausible mechanism for the formation of ferrocenyl bis-amides **1**–**10**.

As shown in Table 1, diverse aromatic aldehydes, involving ferrocenealdehyde, with an electron-rich or electron-deficient group could afford the desired products in high yields. In most cases, the types of amines and isocyanides exhibited less effect on the reaction. Based on the known results [14,15,23], a possible mechanism (Scheme 2) was suggested for the formation of ferrocenyl bis-amides **1**–**10**: firstly, the reaction of amine with aldehyde yields an imine **I**, which further interacts with ferrocenecarboxylic acid to give an imine salt **II**; secondly, it reacts with isocyanide to form an intermediate product **III**, which then rearranges into the final product bis-amide via an acyl transfer process (Mumm rearrangement). It should be noted that the U-4CR strategy provides an efficient synthesis of novel dipeptide-like molecules containing one or two ferrocenyl moieties. This is the simplest and greenest method for the construction of different types of ferrocenyl bis-amide libraries.

Ferrocenyl bis-amides **1**–**10** are yellow solids and stable in the air. Their chemical structures were fully confirmed by IR, NMR and HR-MS spectra. In the IR spectra, products **1**–**10** exhibited two absorption bands at 1666–1680 and 1600–1611 cm^−1^, respectively, ascribed to two C=O groups of bis-amide. Moreover, absorption peaks of the amide NH groups were observed at 3290–3386 cm^−1^. The ^1^H-NMR spectra elucidated the singlet signals for methyl, *t*-butyl and methoxy groups, as well as the N–CH moieties. The phenyl protons showed broad resonances at δ_H_ 7.24–6.71 ppm. Interestingly, for the C_5_H_4_ ring of the ferrocenyl unit linked to amide group, four distinct signals at δ_H_ 4.33–3.56 ppm were found rather than the typically observed multiplets. This indicates that the CH groups at the C_5_H_4_ ring are diastereotopic.

### 2.2. Crystal Structures

**Figure 1:**
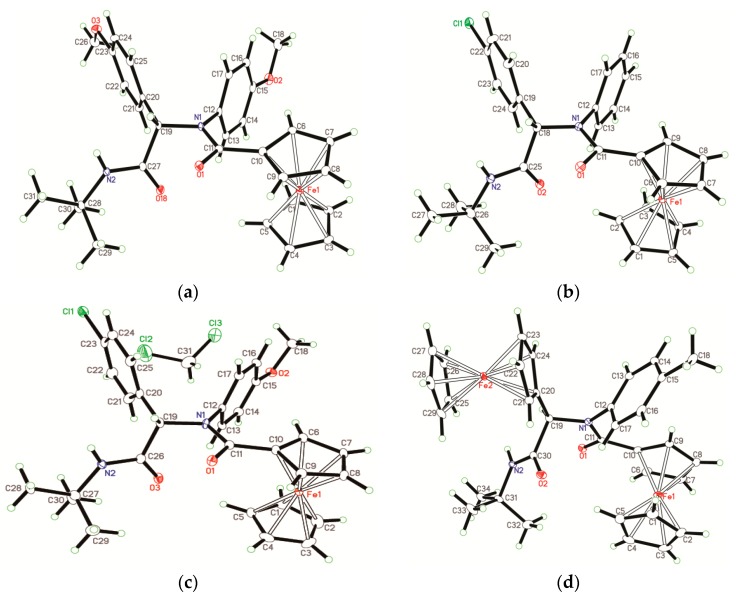
Crystal structures of **3** (**a**), **4** (**b**), **6** (**c**), and **9** (**d**), showing the atom-labelling scheme. Displacement ellipsoids are drawn at the 30% probability level.

To evaluate the precise morphology of these new ferrocenyl bis-amide molecules, the crystal structures of typical products **3**, **4**, **6**, and **9** (Figure 1) were further identified by X-ray diffraction analysis. Their crystal data involving structure refinement (Table S1), bond lengths and bond angles are summarized in the Supplementary Materials (Tables S2–S5). Table 2 and Table 3 give some torsion/dihedral angles, and hydrogen bond geometry, respectively.

The geometric parameters of **3**, **4**, **6**, and **9** are very similar to those of ferrocenyl bis-amides described previously [16]. The crystal analysis indicated that there exist two C=O groups in the bis-amide chain, which is consistent with the characteristic stretching frequency in the IR spectra. Molecules **3** and **9** crystallize in the space group *P*-1, while **4** and **6** take in the space group *P*2_1/n_. In the case of **6**, one dichloromethane solvent molecule is found in its crystal structure, fixed by different C−H⋅⋅⋅Cl interactions (Table 3).

In the solid state, as shown in Table 2, two cyclopentadienyl (Cp) rings of all ferrocenyl groups in four compounds give a nearly eclipsed fashion with a narrow torsion angle range (less than 1°), while they locate almost parallel to each other, with a mean dihedral angle range of 0.5(2)°–0.9(1)°. In the bis-amide chain, two amide C=O groups of each molecule are perpendicular mutually and produce dihedral angles from 78.0(2)° to 84.6(3)°. The amide group lies nearly coplanar with its linked Cp ring, yielding a conjugated system with dihedral angles of 1.0(3)°−13.1(2)°, but it is almost perpendicular to its connected phenyl ring with dihedral angles of 82.4(2)°–89.5(3)°, due to the existence of an intramolecular C−H⋅⋅⋅π interaction [24,25,26] between the C_5_H_4_ ring and its vicinal phenyl ring bonded to N atom (Table 3). This results in the bond length between the N atom and C=O group falling in the shortest one among three N−C bonds. In addition, such an intramolecular C−H⋅⋅⋅π interaction leads the CH groups at the C_5_H_4_ ring to be diastereotopic, which further rationalizes the characters in their NMR spectra. The other amide group displays normal geometric parameters [27].

**Figure 2:**
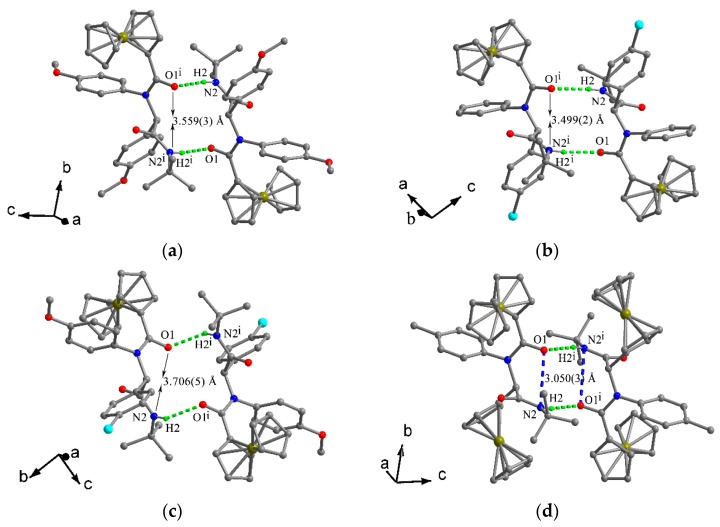
Dimers of compounds **3** (**a**), **4** (**b**), **6** (**c**), and **9** (**d**), showing a 14-membered ring motif.

In the packing, these molecules all create a centro-symmetric dimer by co-operative hard N−H⋅⋅⋅O hydrogen bonds between amide groups (Table 3), generating a 14-membered ring motif (Figure 2). In the ring motifs of **3**, **4**, and **6**, the separations of intra- and intermolecular N and O atoms rank from 3.499(2)−3.706(5) and 2.968(5)−3.037(3) Å, respectively. In the case of **9**, remarkably, the corresponding distances of intramolecular N and O atoms are only 3.050(3) Å, much shorter than those of **3**, **4**, and **6**. This may be ascribed to the steric effect of the second ferrocenyl unit introduced. In addition, these dimers all assemble into molecular chains in the crystal packing, which further associate into supramolecular networks by a combination of different intermolecular contacts (Table 3).

### 2.3. Electrochemistry

**Figure 3:**
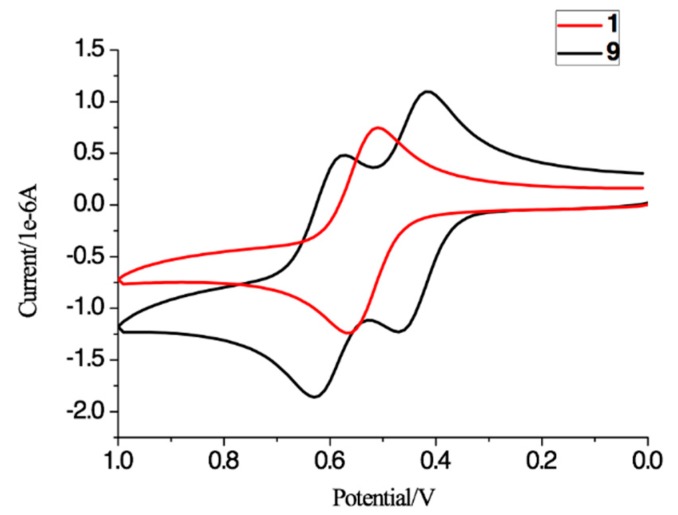
Cyclic voltammograms of **1** and **9** (5.0 × 10^−4^ M) in CH_2_Cl_2_/CH_3_CN (1:1, *v/v*) solution of *n*-Bu_4_NPF_6_ (0.1 M) at a scanning rate of 100 mVs^−1^.

In view of the ferrocenyl incorporated into bis−amides **1**–**10**, their electrochemistry was studied using cyclic voltammetry (CV) and the results are summarized in Table 4. The CV curves are shown in Figure 3 (**1** and **9**) and Figures S1–S8 (**2**–**8**, **10**, see Supplementary Materials). Molecules **1**–**8** exhibit one similar single reversible wave corresponding to the ferrocene/ferrocenium (Fc/Fc^+^) redox couple. In comparison with the parent ferrocene, their *E*_1/2_ values are positively shifted up to ca. 130–150 mV, indicating that these ferrocenyl bis-amides are oxidized more difficultly than the ferrocene owing to the electron-withdrawing character of the amide group [28]. However, in the cases of **9** and **10**, two distinct CV waves originated from two different ferrocenyl moieties were observed at *E*_1/2_ values of 600 and 440 mV, respectively. The former corresponds to the ferrocenyl group linking amide unit, and the latter belongs to the other ferrocenyl group. It is worth noting that the introduction of the second ferrocenyl group can markedly alter the electrochemical behavior of the original one, with a bigger *E*_1/2_ value and less reversibility. In view of these compounds’ incorporation of the redox-active ferrocenyl function and bis-amide chains, they can potentially be applied as electrochemical sensors for selective detection of cationic and anionic substrates.

## 3. Experimental Section

### 3.1. Materials and Instruments

Ferrocenealdehyde and ferrocenecarboxylic acid were prepared according to the literature methods [29,30,31]. Other reagents are commercially available and used without further purification. All solvents except for the separation were purified according to the standard procedures before use.

Infrared (IR) samples were prepared as KBr pellets, and spectra were measured in the 400−4000 cm^−1^ range by a Perkin-Elmer 1600 FTIR spectrometer (Perkin-Elmer, Waltham, MA, USA). ^1^H-NMR and ^13^C-NMR spectra were recorded on a BRUKER AVANCE 300 or BRUKER AVANCE-III HD 400 NMR spectrometer (Bruker, Fällanden, Switzerland, CDCl_3_, TMS as internal standard). Chemical shifts of ^1^H- and ^13^C-NMR spectra are reported in parts per million (ppm). All coupling constants (*J* values) were reported in Hertz (Hz). The following abbreviations were used to explain multiplicities: s = singlet, d = doublet, m = multiplet, and br = broad. High resolution mass spectra (HR-MS) were performed on a maXis UHR-TOF system (Bruker, Daltonics, Germany). Melting points were measured on a Yanaco MP-500 micro melting point apparatus (Yanaco, Kyoto, Japan) and uncorrected. All crystal data were obtained by Agilent SuperNova X-Ray single crystal diffractometer (Agilent, Wroclaw, Poland). Electrochemical experiments were performed with a CHI 660A electrochemical analyzer (CH Instruments, Austin, TX, USA). Column chromatography was performed on silica gel 200–300 mesh. Analytical thin-layer chromatography (TLC) was performed on pre-coated, glass-backed silica gel plates.

### 3.2. Synthesis

General procedure: A solution of ferrocenecarboxylic acid (1.0 mmol) and isocyanide (1.0 mmol) in dry MeOH (1.0 mL) was added to the reacting solution of aryl amine (1.0 mmol) and aldehyde (1.0 mmol) in dry MeOH (2.0 mL) stirred at 45 °C for 2 h. The resulting mixture was continuously stirred at 45 °C for 24 h and then cooled to room temperature. The solvent was evaporated under reduced pressure and the residue was subjected to flash column chromatography (silica gel, ethyl acetate/hexane = 1:2, *v/v*), giving products **1**–**10**.

*N-tert-Butyl-2-(4-methoxyphenyl)-2-(N-phenylferrocenylformamido)acetamide* (**1**), *R*_f_ = 0.4, yellow solid, yield 73%, m.p. 191–192 °C. IR (KBr, cm^−1^): 3308 (N-H), 1675 (C=O), 1602 (C=O), 1248 (Ar-O-C). ^1^H-NMR (CDCl_3_, 300 MHz): δ 7.24 (br, 5H, Ar-H), 7.17 (d, *J* = 9.00 Hz, 2H, Ar-H), 6.72 (d, *J* = 9.00 Hz, 2H, Ar-H), 5.96 (s, 1H, N-CH), 5.89 (s, 1H, NH), 4.22 (s, 1H, Cp-H), 4.20 (s, 5H, Cp-H), 4.14 (s, 1H, Cp-H), 4.06 (s, 1H, Cp-H), 3.75 (s, 3H, OCH_3_), 3.58 (s, 1H, Cp-H), 1.39 (s, 9H, *t*-Bu); ^13^C-NMR (CDCl_3_, 75 MHz): δ 170.64, 169.35, 159.33, 140.94, 131.51, 131.01, 128.63, 127.92, 127.02, 113.60, 75.23, 72.15, 70.94, 70.37, 70.12, 69.83, 66.80, 55.15, 51.38, 28.69. HR–MS (ESI), calcd. for C_30_H_32_FeN_2_O_3_: [M + H]^+^ 525.1841, found: 525.1862 ([M + H]^+^).

*N-tert-Butyl-2-(4-methoxyphenyl)-2-[N-(4-methylphenyl)ferrocenylformamido]acetamide* (**2**), *R*_f_ = 0.4, yellow solid, yield 68%, m.p. 215–216 °C. IR (KBr, cm^−1^): 3290 (N-H), 1676 (C=O), 1609 (C=O), 1246 (Ar−O−C). ^1^H-NMR (CDCl_3_, 300 MHz): δ 7.17 (d, *J* = 9.00 Hz, 2H, Ar-H), 7.04 (br, 4H, Ar-H), 6.74 (d, *J* = 9.00 HZ, 2H, Ar-H), 5.93 (s, 2H, NH, N-CH), 4.20 (s, 6H, Cp-H), 4.14 (s, 1H, Cp-H), 4.06 (s, 1H, Cp-H), 3.76 (s, 3H, OCH_3_), 3.63 (s, 1H, Cp-H), 2.32 (s, 3H, CH_3_), 1.38 (s, 9H, *t*-Bu); ^13^C-NMR (CDCl_3_, 75 MHz): δ 170.75, 169.40, 159.27, 138.25, 137.82, 131.52, 130.62, 129.32, 127.15, 113.57, 75.19, 72.17, 71.06, 70.37, 70.13, 69.81, 66.87, 55.17, 51.35, 28.69, 21.19. HR–MS (ESI), calcd. for C_31_H_34_FeN_2_O_3_: [M + H]^+^ 539.1997, found: 539.2019 ([M + H]^+^).

*N-tert-Butyl-2-(4-methoxyphenyl)-2-[N-(4-methoxyphenyl)ferrocenylformamido]acetamide* (**3**), *R*_f_ = 0.3, yellow solid, yield 81%, m.p. 186–187 °C. IR (KBr, cm^−1^): 3321 (N-H), 1679 (C=O), 1607 (C=O), 1243 (Ar−O−C). ^1^H-NMR (CDCl_3_, 300 MHz): δ 7.15–7.12 (m, 3H, Ar-H), 6.74–6.71 (m, 5H, Ar-H), 5.99 (s, 1H, N-CH), 5.86 (s, 1H, NH), 4.25 (s, 1H, Cp-H), 4.20 (s, 5H, Cp-H), 4.15 (s, 1H, Cp-H), 4.07 (s, 1H, Cp-H), 3.79 (s, 3H, OCH_3_), 3.75 (s, 3H, OCH_3_), 3.58 (s, 1H, Cp-H), 1.38 (s, 9H, *t*-Bu); ^13^C-NMR (CDCl_3_, 75 MHz): δ 170.86, 169.49, 159.28, 159.02, 133.34, 132.16, 131.66, 127.05, 113.67, 113.57, 75.08, 72.27, 70.98, 70.49, 70.21, 69.78, 66.26, 55.34, 55.17, 51.36, 28.69. HR–MS (ESI), calcd. for C_31_H_34_FeN_2_O_4_: [M + H]^+^ 555.1946, found: 555.1975 ([M + H]^+^).

*N-tert-Butyl-2-(4-chlorophenyl)-2-(N-phenylferrocenylformamido)acetamide* (**4**), *R*_f_ = 0.3, yellow solid, yield 73%, m.p. 206–207 °C. IR (KBr, cm^−1^): 3305 (N-H), 1674 (C=O), 1600 (C=O). ^1^H-NMR (CDCl_3_, 300 MHz): δ 7.20 (br, 9H, Ar-H), 6.02 (s, 1H, NH), 5.96 (s, 1H, N-CH), 4.22 (s, 1H, Cp-H), 4.20 (s, 5H, Cp-H), 4.16 (s, 1H, Cp-H), 4.07 (s, 1H, Cp-H), 3.59 (s, 1H, Cp-H), 1.40 (s, 9H, *t*-Bu); ^13^C-NMR (CDCl_3_, 100 MHz): δ 170.78, 168.82, 140.72, 134.16, 133.52, 131.54, 130.88, 128.91, 128.44, 128.27, 75.06, 72.40, 71.18, 70.90, 70.64, 70.11, 66.70, 51.57, 28.68. HR–MS (ESI), calcd. for C_29_H_29_ClFeN_2_O_2_: [M + H]^+^ 529.1345, found: 529.1325 ([M + H]^+^).

*N-tert-Butyl-2-(4-chlorophenyl)-2-[N-(4-methylphenyl)ferrocenylformamido]acetamide* (**5**), *R*_f_ = 0.5, yellow solid, yield 91%, m.p. 228–229 °C. IR (KBr, cm^−1^): 3297 (N-H), 1672 (C=O), 1611 (C=O). ^1^H-NMR (CDCl_3_, 300 MHz): δ 7.20 (br, 5H, Ar-H), 7.04 (br, 3H, Ar-H), 6.06 (s, 1H, NH), 5.93 (s, 1H, N-CH), 4.19–4.16 (m, 7H, Cp-H), 4.08 (s, 1H, Cp-H), 3.63 (s, 1H, Cp-H), 2.34 (s, 3H, CH_3_), 1.39 (s, 9H, *t*-Bu); ^13^C-NMR (CDCl_3_, 100 MHz): δ 171.10, 168.85, 138.23, 138.03, 134.07, 133.65, 131.57, 130.47, 129.57, 128.40, 75.01, 72.43, 71.39, 70.98, 70.60, 69.87, 66.76, 51.52, 28.69, 21.23. HR–MS (ESI), calcd. for C_30_H_31_ClFeN_2_O_2_: [M + H]^+^ 543.1502, found: 543.1497 ([M + H]^+^).

*N-tert-Butyl-2-(4-chlorophenyl)-2-[N-(4-methoxyphenyl)ferrocenylformamido]acetamide* (**6**), *R*_f_ = 0.5, yellow solid, yield 76%, m.p. 173–174 °C. IR (KBr, cm^−1^): 3312 (N-H), 1680 (C=O), 1608 (C=O), 1245 (Ar−O−C). ^1^H-NMR (CDCl_3_, 300 MHz): δ 7.18 (br, 5H, Ar-H), 6.77–6.74 (m, 3H, Ar-H), 5.99 (s, 2H, NH, N-CH), 4.25 (s, 1H, Cp-H), 4.19 (s, 5H, Cp-H), 4.17 (s, 1H, Cp-H), 4.09 (s, 1H, Cp-H), 3.80 (s, 3H, OCH_3_), 3.58 (s, 1H, Cp-H), 1.39 (s, 9H, *t*-Bu); ^13^C-NMR (CDCl_3_, 100 MHz): δ 171.00, 169.02, 159.25, 134.12, 133.57, 133.08, 132.13, 131.73, 128.41, 113.91, 75.11, 72.40, 71.17, 70.91, 70.60, 69.97, 66.11, 55.41, 51.53, 28.69. HR–MS (ESI), calcd. for C_30_H_31_ClFeN_2_O_3_: [M + H]^+^ 559.1451, found: 559.1444 ([M + H]^+^).

*N-Cyclohexyl-2-(4-methoxyphenyl)-2-[N-(4-methylphenyl)ferrocenylformamido]acetamide* (**7**), *R*_f_ = 0.4, yellow solid, yield 87%, m.p. 164–165 °C. IR (KBr, cm^−1^): 3386 (N-H), 1674 (C=O), 1608 (C=O), 1237 (Ar−O−C). ^1^H-NMR (CDCl_3_, 400 MHz): δ 7.15–7.13 (m, 3H, Ar-H), 7.04–7.03 (m, 3H, Ar-H), 6.74 (d, *J* = 8.00 Hz, 2H, Ar-H), 6.08 (s, 1H, N-CH), 5.86 (d, *J* = 4.00 Hz, 1H, NH), 4.23 (s, 1H, Cp-H), 4.19 (s, 5H, Cp-H), 4.14 (s, 1H, Cp-H), 4.06 (s, 1H, Cp-H), 3.76 (s, 3H, OCH_3_), 3.59 (s, 1H, Cp-H), 2.33 (s, 3H, CH_3_), 2.00–1.06 (m, 11H, cychexyl-H); ^13^C-NMR (CDCl_3_, 100 MHz): δ 170.82, 169.13, 159.38, 138.22, 137.94, 131.67, 130.74, 129.38, 127.18, 113.64, 75.37, 72.41, 71.26, 70.75, 70.47, 70.08, 66.22, 55.21, 48.62, 32.96, 32.90, 25.57, 24.87, 24.79, 21.23. HR–MS (ESI), calcd. for C_33_H_36_FeN_2_O_3_: [M + H]^+^ 565.2154, found: 565.2160 ([M + H]^+^).

*N-Cyclohexyl-2-(4-methoxyphenyl)-2-[N-(4-methoxyphenyl)ferrocenylformamido]acetamide* (**8**), *R*_f_ = 0.5, yellow solid, yield 91%, m.p. 167–168 °C. IR (KBr, cm^−1^): 3293 (N-H), 1672 (C=O), 1608 (C=O), 1237 (Ar−O−C). ^1^H-NMR (CDCl_3_, 400 MHz): δ 7.11–7.09 (m, 3H, Ar-H), 6.74–6.71 (m, 5H, Ar-H), 6.15 (s, 1H, N-CH), 5.82 (d, *J* = 4.00 Hz, 1H, NH), 4.28 (s, 1H, Cp-H), 4.22 (s, 5H, Cp-H), 4.17 (s, 1H, Cp-H), 4.09 (s, 1H, Cp-H), 3.79 (s, 3H, OCH_3_), 3.75 (s, 3H, OCH_3_), 3.58 (s, 1H, Cp-H), 2.00–1.02 (m, 11H, cychexyl-H); ^13^C-NMR (CDCl_3_, 100 MHz): δ 170.91, 169.20, 159.40, 159.12, 133.32, 132.27, 131.78, 127.13, 113.71, 113.64, 75.25, 72.36, 71.07, 70.66, 70.33, 69.91, 65.66, 55.37, 55.19, 48.62, 32.94, 32.87, 25.56, 24.86, 24.78. HR–MS (ESI), calcd. for C_33_H_36_FeN_2_O_4_: [M + H]^+^ 581.2103, found: 581.2111 ([M + H]^+^).

*N-tert-Butyl-2-ferrocenyl-2-[N-(4-methylphenyl)ferrocenylformamido]acetamide* (**9**), *R*_f_ = 0.5, yellow solid, yield 87%, m.p. 219–220 °C. IR (KBr, cm^−1^): 3323 (N-H), 1666 (C=O), 1608 (C=O). ^1^H-NMR (CDCl_3_, 300 MHz): δ 7.03–7.01 (m, 3H, N-H, Ar-H), 6.85 (br, 2H, Ar-H), 5.95 (s, 1H, N-CH), 4.33 (s, 1H, Cp-H), 4.16–4.08 (m, 14H, Cp-H), 4.03 (s, 2H, Cp-H), 3.72 (s, 1H, Cp-H), 2.32 (s, 3H, CH_3_), 1.49 (s, 9H, *t*-Bu); ^13^C-NMR (CDCl_3_, 100 MHz): δ 170.67, 168.37, 138.02, 137.23, 130.47, 129.10, 81.37, 75.08, 72.08, 71.44, 71.32, 70.49, 70.39, 69.82, 69.39, 69.03, 68.41, 51.11, 28.83, 21.27. HR–MS (ESI), calcd. for C_34_H_36_Fe_2_N_2_O_2_: [M + H]^+^ 617.1554, found: 617.1554 ([M + H]^+^).

*N-tert-Butyl-2-ferrocenyl-2-[N-(4-methoxyphenyl)ferrocenylformamido]acetamide* (**10**), *R*_f_ = 0.6, yellow solid, yield 91%, m.p. 180–181 °C. IR (KBr, cm^−1^): 3297 (N-H), 1668 (C=O), 1607 (C=O), 1239 (Ar−O−C). ^1^H-NMR (CDCl_3_, 300 MHz): δ 6.80–6.74 (m, 5H, NH, Ar-H), 5.97 (s, 1H, N-CH), 4.31 (s, 1H, Cp-H), 4.13–4.08 (m, 15H, Cp-H), 4.03 (s, 1H, Cp-H), 3.79 (s, 3H, OCH_3_), 3.70 (s, 1H, Cp-H), 1.50 (s, 9H, *t*-Bu); ^13^C-NMR (CDCl_3_, 100 MHz): δ 170.69, 168.45, 159.24, 132.38, 131.89, 113.50, 81.32, 74.94, 72.16, 71.24, 70.59, 70.46, 69.86, 69.77, 69.38, 69.18, 68.25, 55.37, 51.12, 28.83. HR–MS (ESI), calcd. for C_34_H_36_Fe_2_N_2_O_3_: [M + H]^+^ 633.1503, found: 633.1507 ([M + H]^+^).

### 3.3. Crystal Structure Determination

Single crystals of **3**, **4**, **6**, and **9** suitable for X-ray diffraction analysis were obtained by slow evaporation of their solution in dichloromethane–hexane (1:1, *v/v*) at 0 °C. Selected light orange single crystals of **3**, **4**, **6**, and **9** were mounted on glass fibers, respectively. The intensity data were measured at 293 K (**3**) and 100 K (**4**, **6**, and **9**) on an Agilent SuperNova CCD-based diffractometer (Cu Kα radiation, λ = 1.54184 Å). Empirical absorption corrections were applied using SCALE3 ABSPACK. The structures were solved by direct methods and difference Fourier syntheses, and refined by full-matrix least-squares technique on F2 using SHELXS-97 [32] and SHELXL-97 [33]. All non-hydrogen atoms were refined with anisotropic displacement parameters. Hydrogen atoms attached to refined atoms were placed in geometrically idealized positions and refined using a riding model with C–H = 0.93, 0.97 and 0.96 Å for aromatic, methylene and methyl H, respectively, Uiso(H) = 1.5 Ueq(C) for methyl H, and Uiso(H) = 1.2 Ueq(C) for all other H atoms. Crystallographic data for the structures **3**, **4**, **6**, and **9** have been deposited in the Cambridge Crystallography Data Centre (CCDC No. 1536917, 1536922, 1536932 and 1536933, respectively).

### 3.4. Electrochemistry Studies

Cyclic voltametry measurements were performed using a solution of products **1**–**10** (5.0 × 10^−4^ M) in CH_2_Cl_2_/CH_3_CN (1:1, *v/v*) solution with 0.1 M *n*-Bu_4_NPF_6_ as supporting electrolyte, Hg/Hg_2_Cl_2_ as the reference electrode, along with platinum working and auxiliary electrodes, and a scan rate of 100 mVs^−1^.

## 4. Conclusions

In conclusion, the scope of constructing novel ferrocenyl bis-amides via the U-4CR strategy has been assessed, whereby ten novel ferrocenyl bis-amides were successfully synthesized and fully characterized by IR, NMR, HR-MS, X-ray diffraction, and electrochemical analyses. This method features a high degree of synthetic efficiency and atom economy. It is very useful for constructing ferrocenyl peptide-like molecule libraries that can be potentially applied electrochemical sensors in biological and environmental systems.

## Figures and Tables

**Table 1:** Synthesis of ferrocenyl bis-amides **1**–**10**.

Product	RNH_2_	R′CHO	R″NC	Yield (%)
**1**	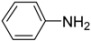	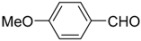	*t*-BuNC	73
**2**	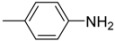	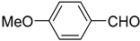	*t*-BuNC	68
**3**	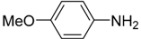	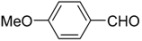	*t*-BuNC	81
**4**	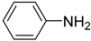	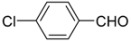	*t*-BuNC	73
**5**	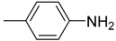	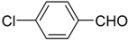	*t*-BuNC	91
**6**	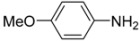	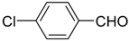	*t*-BuNC	76
**7**	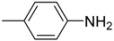	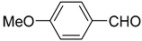	CyNC	87
**8**	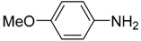	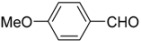	CyNC	91
**9**	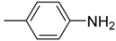	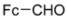	*t*-BuNC	87
**10**	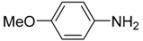	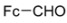	*t*-BuNC	91

**Table 2:** Some torsion or dihedral angles (°) for **3**, **4**, **6**, and **9**.

	3	4	6	9	
*T*_1_	−5.4(2)	4.7(1)	−0.3(3)	−1.1(2)	1.1(2)
*T*_2_	−6.0(2)	4.9(1)	−10.2(4)	−0.8(2)	0.8(2)
*T*_3_	−5.8(2)	4.5(1)	−10.1(4)	−1.2(2)	1.2(2)
*T*_4_	−5.5(2)	4.5(1)	−10.3(4)	−0.8(2)	0.8(2)
*T*_5_	−5.4(2)	4.0(1)	−10.9(3)	−1.7(2)	1.7(2)
*D*_1_	0.7(1)	0.9(1)	0.5(2)	0.7(1)	
*D*_2_	78.0(2)	83.2(2)	80.8(4)	84.6(3)	
*D*_3_	13.1(2)	3.0(1)	1.0(3)	7.9(2)	
*D*_4_	82.4(2)	87.4(2)	89.5(3)	83.3(2)	

*T*_1-5_ are torsion angles between two cyclopentadienyl (Cp) rings of the ferrocenyl unit, viz. C−*Cg1*−*Cg2*−C (*Cg*1 and *Cg*2 are the centroids of two Cp rings.); *D*_1_ and *D*_2_ are dihedral angles of two Cp rings and two amide planes, respectively; *D*_3_ and *D*_4_ are dihedral angles of the amide plane with its connected Cp ring and phenyl ring, respectively.

**Table 3:** Hydrogen bond geometry (Å,°) for **3**, **4**, **6** and **9**.

Product	D−H⋅⋅⋅A	D−H	H⋅⋅⋅A	D⋅⋅⋅A	D−H⋅⋅⋅A
**3**	C6−H6⋅⋅⋅*Cg*3	0.93	2.69	3.454(2)	138.9
	N2−H2⋅⋅⋅O1 ^a^	0.86	2.16	3.016(3)	171.7
	C14−H14⋅⋅⋅O18 ^b^	0.93	2.38	3.298(3)	169.1
	C22−H22⋅⋅⋅*Cg*1 ^b^	0.93	2.82	3.627(3)	145.3
**4**	C9−H9⋅⋅⋅*Cg*3	0.93	2.60	3.392(2)	142.9
	N2−H2⋅⋅⋅O1 ^a^	0.86	2.18	3.037(3)	173.6
	C17−H17⋅⋅⋅ *Cg*1 ^b^	0.93	2.99	3.674(2)	131.9
**6**	C6−H6⋅⋅⋅*Cg*3	0.98	2.44	3.275(5)	142.5
	N2−H2⋅⋅⋅O1 ^a^	0.86	2.22	2.968(5)	145.0
	C19–H19··· O1 ^a^	0.98	2.41	3.245(5)	142.5
	C8–H8···O3 ^b^	0.98	2.23	3.198(5)	169.1
	C4–H4···Cl3 ^c^	0.98	2.59	3.529(6)	160.8
	C31–H31A···Cl3 ^d^	0.97	2.74	3.489(8)	134.7
**9**	C9–H9···*Cg*3	0.93	2.52	3.308(2)	143.0
	N2–H2···O1 ^a^	0.86	2.16	3.009(3)	169.8

Symmetry codes: **3**, (a) −x, 2 − y, 1 – z; (b) −x, 2 − y, −z. **4**, (a) 2 − x, 2 − y, 1 − z; (b) x, 1 + y, z. **6**, (a) 1 − x, −y, 1 − z; (b) 1/2 − x, −1/2 + y, 1/2 − z; (c) −1/2 + x, 1/2 − y, 1/2 + z; (d) 1 − x, 1 − y, −z. **9**, (a) −x, 1 − y, 1 − z. Cg1 is the centroid of the C1−C5 ring for **3** and **4**, Cg3 is the centroid of the C12−C17 ring for **3**, **4**, **6** and **9**.

**Table 4:** Electrochemical properties of ferrocenyl bis-amides **1**–**10** ^a^.

Product	*E*_pa_ (mV)	*E*_pc_ (mV)	*E*_pa_ − *E*_pc_ (mV)	*E*_1/2_ (mV)	*I*_pa_/*I*_pc_
**1**	570	510	60	540	1.02
**2**	560	500	60	530	1.04
**3**	560	500	60	530	1.01
**4**	580	520	60	550	1.04
**5**	580	520	60	550	1.05
**6**	580	520	60	550	1.04
**7**	560	500	60	530	1.03
**8**	560	500	60	530	1.00
**9** ^b^	630, 470	570, 410	60, 60	600, 440	1.58, 1.04
**10** ^b^	630, 470	570, 410	60, 60	600, 440	1.51, 1.06

^a^ Conditions: 5.0 × 10^−4^ M of **1**–**10** and 0.1 M *n*-Bu_4_NPF_6_ in CH_2_Cl_2_/CH_3_CN (1:1, *v/v*), Pt disk working electrode, Pt auxiliary electrode, Hg/Hg_2_Cl_2_ reference electrode and scan rate at 100 mVs^−1^. Errors: ± 10 mV; ^b^ The left *E*_1/2_ value is ascribed to the ferrocenyl moiety linking the amide group and the right one to the other ferrocenyl group.

## Supplementary Materials

The following are available online, 1. Crystal data and structural refinement for **3**, **4**, **6**, and **9**; 2. Bond lengths and bond angles for **3**, **4**, **6**, and **9**; 3. Cyclic voltammograms for **2**–**8** and **10**; 4. NMR Spectra.
