# Supplementary material for: Synthesis, Crystal Structures and Properties of Ferrocenyl Bis-Amide Derivatives Yielded via the Ugi Four-Component Reaction
Source: Molecules. 2017 May 4;22(5):737. doi: 10.3390/molecules22050737 (PMC6154595; doi:10.3390/molecules22050737)

Supplementary Materials

for

Synthesis, crystal structures and properties of ferrocenyl bis-amide derivatives generated via the Ugi four-component reaction

Mei Zhao, Guang-Kui Shao, Dan-Dan Huang, Xue-Xin Lv and Dian-Shun Guo*

College of Chemistry, Chemical Engineering and Materials Science, Collaborative Innovation Center of Functionalized Probes for Chemical Imaging in Universities of Shandong, Shandong Normal University, Jinan 250014, China.

* Author to whom correspondence should be addressed; e-mail: chdsguo@sdnu.edu.cn;
Tel.: +86-531-86180743; Fax: +86-531-86928773.

Contents

1. Crystal data and structural refinement for **3**, **4**, **6**, and **9**……3

2. Bond lengths and bond angles for **3**, **4**, **6**, and **9**……3

3. Cyclic voltammograms for **2**-**8** and **10**……14

4. NMR Spectra…… 18

1. Crystal data and structural refinement for **3**, **4**, **6**, and **9**

Table S1. Crystal data and structural refinement for **3**, **4**, **6**, and **9**.

Code	**3**	**4**	**6**	**9**
Empirical formula	$C_{31}H_{34}FeN_2O_4$	$C_{29}H_{29}ClFeN_2O_2$	$C_{30}H_{31}ClFeN_2O_3{\cdot}CH_2Cl_2$	$C_{34}H_{36}Fe_2N_2O_2$
Formula weight	554.45	528.84	643.79	616.35
Temperature (K)	293	100	100	100
Crystal system	Triclinic	Monoclinic	Monoclinic	Triclinic
Space group	*P* -1	$P2_1/n$	$P2_1/n$	*P* -1
a (Å)	10.6830(4)	14.7919(10)	14.1536(13)	10.3246(5)
b (Å)	11.8625(5)	9.3913(7)	14.2437(11)	11.1236(7)
c (Å)	12.1862(4)	18.8165(14)	14.7080(13)	13.4646(7)
α (°)	102.295(3)	90.00	90.00	76.795(5)
β (°)	104.448(3)	102.613(7)	96.441(8)	89.125(4)
γ (°)	109.372(4)	90.00	90.00	71.173(5)
Volume (Å^3)	1334.86(9)	2550.8(3)	2946.4(4)	1422.04(13)
Z	2	4	4	2
D_c (mg/m^3)	1.379	1.377	1.451	1.439
μ (mm^{-1})	0.61	0.73	0.82	1.05
F(000)	584	1104	1336	644
Crystal size (mm)	0.11×0.08×0.04	0.19×0.06×0.02	0.17×0.07×0.02	0.25×0.05×0.04
θ range (°)	2.96 to 25.00	3.19 to 25.00	3.13 to 25.68	3.83 to 28.99
Collected reflections	14931	15282	14122	15777
Independent reflections	4701	4479	5585	4995
Observed reflections [$I > 2\sigma(I)$]	4069	3849	4329	4215
Goodness-of-fit on F^2	1.111	1.072	1.034	1.047
Final *R* indices [$I > 2\sigma(I)$]	*R* = 0.0377,	*R* = 0.0319,	*R* = 0.0705,	*R* = 0.0342,
	w*R* = 0.0817	w*R* = 0.0711	w*R* = 0.1978	w*R* = 0.0738
R indices (all data)	*R* = 0.0472,	*R* = 0.0410,	*R* = 0.0919,	*R* = 0.0445,
	w*R* = 0.0868	w*R* = 0.0769	w*R* = 0.2209	w*R* = 0.0810
$(\Delta\rho)_{max}$, $(\Delta\rho)_{min}$ (e·Å^{-3})	0.444, -0.435	0.310, -0.316	1.310, -1.644	0.348, -0.409

2. Bond lengths and bond angles for **3**, **4**, **6**, and **9**

Table S2. Bond lengths (Å) and bond angles (°) for **3**.

Fe1—C5	2.046 (2)	C27—C19	1.549 (3)
Fe1—C1	2.050 (2)	C20—C21	1.385 (3)
Fe1—C9	2.051 (2)	C20—C25	1.393 (3)
Fe1—C6	2.051 (2)	C20—C19	1.519 (3)
Fe1—C4	2.051 (3)	C17—C12	1.382 (3)
Fe1—C8	2.057 (2)	C17—C16	1.398 (3)
Fe1—C10	2.058 (2)	C28—C29	1.526 (4)
Fe1—C2	2.061 (3)	C28—C31	1.527 (3)
Fe1—C7	2.061 (2)	C28—C30	1.531 (4)
Fe1—C3	2.063 (2)	C22—C23	1.387 (4)
O1—C11	1.238 (3)	C22—C21	1.390 (4)
O3—C23	1.370 (3)	C12—C13	1.393 (3)
O3—C26	1.433 (3)	C23—C24	1.395 (4)
O2—C15	1.374 (3)	C24—C25	1.386 (4)
O2—C18	1.423 (3)	C15—C16	1.391 (4)
O18—C27	1.227 (3)	C15—C14	1.391 (4)
N2—C27	1.345 (3)	C6—C7	1.421 (3)
N2—C28	1.483 (3)	C4—C3	1.425 (4)
N1—C11	1.366 (3)	C4—C5	1.431 (4)

N1—C12	1.442 (3)	C8—C7	1.416 (4)
N1—C19	1.490 (3)	C13—C14	1.387 (3)
C9—C8	1.418 (4)	C1—C2	1.426 (4)
C9—C10	1.437 (3)	C1—C5	1.429 (4)
C10—C6	1.445 (3)	C2—C3	1.424 (4)
C10—C11	1.489 (3)		
C5—Fe1—C1	40.84 (10)	O18—C27—N2	125.5 (2)
C5—Fe1—C9	120.81 (10)	O18—C27—C19	122.0 (2)
C1—Fe1—C9	156.38 (10)	N2—C27—C19	112.4 (2)
C5—Fe1—C6	125.71 (10)	O1—C11—N1	119.9 (2)
C1—Fe1—C6	108.13 (10)	O1—C11—C10	118.2 (2)
C9—Fe1—C6	68.53 (10)	N1—C11—C10	121.6 (2)
C5—Fe1—C4	40.87 (10)	C21—C20—C25	118.0 (2)
C1—Fe1—C4	68.53 (10)	C21—C20—C19	121.7 (2)
C9—Fe1—C4	107.39 (10)	C25—C20—C19	120.3 (2)
C6—Fe1—C4	162.86 (10)	C12—C17—C16	120.6 (2)
C5—Fe1—C8	155.62 (11)	N2—C28—C29	110.1 (2)
C1—Fe1—C8	162.00 (11)	N2—C28—C31	106.4 (2)
C9—Fe1—C8	40.39 (10)	C29—C28—C31	109.4 (2)
C6—Fe1—C8	68.06 (10)	N2—C28—C30	109.8 (2)
C4—Fe1—C8	120.37 (10)	C29—C28—C30	110.7 (2)
C5—Fe1—C10	107.43 (10)	C31—C28—C30	110.4 (2)
C1—Fe1—C10	121.01 (10)	C23—C22—C21	119.4 (2)
C9—Fe1—C10	40.95 (9)	C17—C12—C13	119.8 (2)
C6—Fe1—C10	41.16 (10)	C17—C12—N1	120.4 (2)
C4—Fe1—C10	124.94 (10)	C13—C12—N1	119.8 (2)
C8—Fe1—C10	68.56 (10)	O3—C23—C22	124.4 (2)
C5—Fe1—C2	68.41 (11)	O3—C23—C24	116.0 (2)
C1—Fe1—C2	40.58 (10)	C22—C23—C24	119.5 (2)
C9—Fe1—C2	161.45 (10)	N1—C19—C20	113.10 (19)
C6—Fe1—C2	121.06 (10)	N1—C19—C27	109.90 (19)
C4—Fe1—C2	68.12 (10)	C20—C19—C27	109.97 (19)
C8—Fe1—C2	124.99 (10)	C25—C24—C23	120.1 (2)
C10—Fe1—C2	156.33 (10)	C24—C25—C20	121.0 (2)
C5—Fe1—C7	162.76 (10)	O2—C15—C16	124.3 (2)
C1—Fe1—C7	125.60 (11)	O2—C15—C14	115.4 (2)
C9—Fe1—C7	67.98 (10)	C16—C15—C14	120.4 (2)
C6—Fe1—C7	40.43 (10)	C7—C6—C10	108.3 (2)
C4—Fe1—C7	155.11 (10)	C7—C6—Fe1	70.16 (14)
C8—Fe1—C7	40.22 (10)	C10—C6—Fe1	69.66 (14)
C10—Fe1—C7	68.63 (10)	C3—C4—C5	108.2 (2)
C2—Fe1—C7	108.05 (10)	C3—C4—Fe1	70.18 (14)
C5—Fe1—C3	68.51 (10)	C5—C4—Fe1	69.36 (14)
C1—Fe1—C3	68.32 (10)	C7—C8—C9	108.4 (2)
C9—Fe1—C3	124.63 (10)	C7—C8—Fe1	70.04 (14)
C6—Fe1—C3	155.46 (10)	C9—C8—Fe1	69.57 (14)
C4—Fe1—C3	40.53 (10)	C15—C16—C17	119.1 (2)
C8—Fe1—C3	107.39 (10)	C20—C21—C22	122.0 (2)
C10—Fe1—C3	161.81 (10)	C8—C7—C6	108.3 (2)
C2—Fe1—C3	40.39 (10)	C8—C7—Fe1	69.74 (14)
C7—Fe1—C3	120.50 (10)	C6—C7—Fe1	69.40 (14)
C23—O3—C26	116.6 (2)	C14—C13—C12	120.1 (2)
C15—O2—C18	118.6 (2)	C2—C1—C5	107.9 (2)
C27—N2—C28	126.4 (2)	C2—C1—Fe1	70.11 (14)
C11—N1—C12	124.07 (19)	C5—C1—Fe1	69.41 (14)
C11—N1—C19	115.03 (19)	C3—C2—C1	108.3 (2)
C12—N1—C19	120.10 (18)	C3—C2—Fe1	69.89 (14)
C8—C9—C10	108.5 (2)	C1—C2—Fe1	69.31 (14)

C8—C9—Fe1	70.04 (14)	C13—C14—C15	120.0 (2)
C10—C9—Fe1	69.78 (14)	C1—C5—C4	107.7 (2)
C9—C10—C6	106.5 (2)	C1—C5—Fe1	69.74 (14)
C9—C10—C11	119.4 (2)	C4—C5—Fe1	69.77 (14)
C6—C10—C11	133.5 (2)	C2—C3—C4	107.9 (2)
C9—C10—Fe1	69.27 (14)	C2—C3—Fe1	69.72 (14)
C6—C10—Fe1	69.17 (14)	C4—C3—Fe1	69.29 (14)
C11—C10—Fe1	132.10 (17)		

Table S3. Bond lengths (Å) and bond angles (°) for **4**.

Fe1—C1	2.040 (2)	C12—C13	1.389 (3)
Fe1—C9	2.042 (2)	C5—C1	1.417 (3)
Fe1—C6	2.043 (2)	C5—H5	0.9300
Fe1—C5	2.045 (2)	C26—C27	1.526 (3)
Fe1—C10	2.046 (2)	C26—C29	1.527 (3)
Fe1—C4	2.047 (2)	C26—C28	1.531 (3)
Fe1—C2	2.049 (2)	C16—C17	1.389 (3)
Fe1—C8	2.051 (2)	C16—H16	0.9300
Fe1—C3	2.052 (2)	C14—C13	1.386 (3)
Fe1—C7	2.055 (2)	C14—H14	0.9300
Cl1—C22	1.741 (2)	C1—C2	1.421 (3)
O2—C25	1.228 (3)	C1—H1	0.9300
O1—C11	1.238 (3)	C8—C9	1.419 (3)
N2—C25	1.337 (3)	C8—H8	0.9300
N2—C26	1.476 (3)	C3—C2	1.414 (3)
N2—H2	0.8600	C3—H3	0.9300
N1—C11	1.365 (3)	C18—C25	1.539 (3)
N1—C12	1.438 (3)	C18—H18	0.9800
N1—C18	1.483 (3)	C13—H13	0.9300
C6—C7	1.414 (3)	C17—H17	0.9300
C6—C10	1.438 (3)	C24—C23	1.382 (3)
C6—H6	0.9300	C24—H24	0.9300
C7—C8	1.419 (3)	C9—H9	0.9300
C7—H7	0.9300	C2—H2A	0.9300
C19—C20	1.387 (3)	C21—C20	1.387 (3)
C19—C24	1.392 (3)	C21—H21	0.9300
C19—C18	1.517 (3)	C20—H20	0.9300
C4—C5	1.418 (3)	C23—H23	0.9300
C4—C3	1.423 (3)	C28—H28A	0.9600
C4—H4	0.9300	C28—H28B	0.9600
C22—C23	1.377 (3)	C28—H28C	0.9600
C22—C21	1.379 (3)	C29—H29A	0.9600
C10—C9	1.439 (3)	C29—H29B	0.9600
C10—C11	1.492 (3)	C29—H29C	0.9600
C15—C16	1.384 (3)	C27—H27A	0.9600
C15—C14	1.390 (3)	C27—H27B	0.9600
C15—H15	0.9300	C27—H27C	0.9600
C12—C17	1.387 (3)		
C1—Fe1—C9	156.22 (9)	Fe1—C5—H5	126.1
C1—Fe1—C6	107.52 (9)	O1—C11—N1	119.41 (19)
C9—Fe1—C6	68.90 (9)	O1—C11—C10	118.78 (19)
C1—Fe1—C5	40.60 (9)	N1—C11—C10	121.80 (18)
C9—Fe1—C5	161.56 (9)	N2—C26—C27	106.22 (19)
C6—Fe1—C5	120.95 (9)	N2—C26—C29	109.69 (18)
C1—Fe1—C10	120.54 (9)	C27—C26—C29	110.5 (2)
C9—Fe1—C10	41.23 (8)	N2—C26—C28	109.63 (19)
C6—Fe1—C10	41.17 (8)	C27—C26—C28	109.6 (2)

C5—Fe1—C10	156.08 (9)	C29—C26—C28	111.1 (2)
C1—Fe1—C4	68.12 (9)	C15—C16—C17	120.5 (2)
C9—Fe1—C4	124.64 (9)	C15—C16—H16	119.8
C6—Fe1—C4	156.34 (9)	C17—C16—H16	119.8
C5—Fe1—C4	40.56 (9)	C13—C14—C15	120.3 (2)
C10—Fe1—C4	161.43 (9)	C13—C14—H14	119.9
C1—Fe1—C2	40.67 (9)	C15—C14—H14	119.9
C9—Fe1—C2	120.76 (9)	C5—C1—C2	108.3 (2)
C6—Fe1—C2	124.73 (9)	C5—C1—Fe1	69.91 (13)
C5—Fe1—C2	68.37 (9)	C2—C1—Fe1	70.00 (12)
C10—Fe1—C2	106.87 (9)	C5—C1—H1	125.8
C4—Fe1—C2	68.10 (9)	C2—C1—H1	125.8
C1—Fe1—C8	161.76 (9)	Fe1—C1—H1	125.8
C9—Fe1—C8	40.59 (9)	C7—C8—C9	108.69 (19)
C6—Fe1—C8	68.09 (9)	C7—C8—Fe1	69.95 (12)
C5—Fe1—C8	125.14 (9)	C9—C8—Fe1	69.36 (12)
C10—Fe1—C8	68.66 (9)	C7—C8—H8	125.7
C4—Fe1—C8	108.35 (9)	C9—C8—H8	125.7
C2—Fe1—C8	156.36 (9)	Fe1—C8—H8	126.6
C1—Fe1—C3	68.13 (9)	C2—C3—C4	107.9 (2)
C9—Fe1—C3	107.36 (9)	C2—C3—Fe1	69.70 (12)
C6—Fe1—C3	161.43 (9)	C4—C3—Fe1	69.48 (12)
C5—Fe1—C3	68.34 (9)	C2—C3—H3	126.0
C10—Fe1—C3	124.13 (9)	C4—C3—H3	126.0
C4—Fe1—C3	40.61 (9)	Fe1—C3—H3	126.3
C2—Fe1—C3	40.32 (9)	N1—C18—C19	113.13 (17)
C8—Fe1—C3	121.68 (9)	N1—C18—C25	109.15 (17)
C1—Fe1—C7	124.88 (9)	C19—C18—C25	109.11 (17)
C9—Fe1—C7	68.53 (9)	N1—C18—H18	108.4
C6—Fe1—C7	40.37 (9)	C19—C18—H18	108.4
C5—Fe1—C7	107.95 (9)	C25—C18—H18	108.4
C10—Fe1—C7	68.75 (9)	O2—C25—N2	124.3 (2)
C4—Fe1—C7	121.68 (9)	O2—C25—C18	121.19 (18)
C2—Fe1—C7	161.56 (9)	N2—C25—C18	114.29 (19)
C8—Fe1—C7	40.45 (9)	C14—C13—C12	119.5 (2)
C3—Fe1—C7	156.85 (9)	C14—C13—H13	120.2
C25—N2—C26	123.95 (19)	C12—C13—H13	120.2
C25—N2—H2	118.0	C12—C17—C16	119.4 (2)
C26—N2—H2	118.0	C12—C17—H17	120.3
C11—N1—C12	124.30 (17)	C16—C17—H17	120.3
C11—N1—C18	115.33 (17)	C23—C24—C19	121.0 (2)
C12—N1—C18	119.73 (17)	C23—C24—H24	119.5
C7—C6—C10	108.6 (2)	C19—C24—H24	119.5
C7—C6—Fe1	70.27 (12)	C8—C9—C10	107.85 (19)
C10—C6—Fe1	69.52 (12)	C8—C9—Fe1	70.05 (12)
C7—C6—H6	125.7	C10—C9—Fe1	69.55 (12)
C10—C6—H6	125.7	C8—C9—H9	126.1
Fe1—C6—H6	126.1	C10—C9—H9	126.1
C6—C7—C8	107.99 (19)	Fe1—C9—H9	125.9
C6—C7—Fe1	69.37 (12)	C3—C2—C1	107.94 (19)
C8—C7—Fe1	69.61 (12)	C3—C2—Fe1	69.98 (12)
C6—C7—H7	126.0	C1—C2—Fe1	69.32 (12)
C8—C7—H7	126.0	C3—C2—H2A	126.0
Fe1—C7—H7	126.6	C1—C2—H2A	126.0
C20—C19—C24	118.4 (2)	Fe1—C2—H2A	126.2
C20—C19—C18	120.3 (2)	C22—C21—C20	119.2 (2)
C24—C19—C18	121.3 (2)	C22—C21—H21	120.4
C5—C4—C3	108.21 (19)	C20—C21—H21	120.4

C5—C4—Fe1	69.66 (12)	C21—C20—C19	121.1 (2)
C3—C4—Fe1	69.90 (12)	C21—C20—H20	119.5
C5—C4—H4	125.9	C19—C20—H20	119.5
C3—C4—H4	125.9	C22—C23—C24	119.5 (2)
Fe1—C4—H4	126.1	C22—C23—H23	120.3
C23—C22—C21	120.9 (2)	C24—C23—H23	120.3
C23—C22—Cl1	119.19 (19)	C26—C28—H28A	109.5
C21—C22—Cl1	119.94 (19)	C26—C28—H28B	109.5
C6—C10—C9	106.89 (19)	H28A—C28—H28B	109.5
C6—C10—C11	118.99 (19)	C26—C28—H28C	109.5
C9—C10—C11	134.0 (2)	H28A—C28—H28C	109.5
C6—C10—Fe1	69.32 (12)	H28B—C28—H28C	109.5
C9—C10—Fe1	69.22 (12)	C26—C29—H29A	109.5
C11—C10—Fe1	123.58 (15)	C26—C29—H29B	109.5
C16—C15—C14	119.7 (2)	H29A—C29—H29B	109.5
C16—C15—H15	120.1	C26—C29—H29C	109.5
C14—C15—H15	120.1	H29A—C29—H29C	109.5
C17—C12—C13	120.53 (19)	H29B—C29—H29C	109.5
C17—C12—N1	118.91 (19)	C26—C27—H27A	109.5
C13—C12—N1	120.56 (19)	C26—C27—H27B	109.5
C1—C5—C4	107.6 (2)	H27A—C27—H27B	109.5
C1—C5—Fe1	69.49 (12)	C26—C27—H27C	109.5
C4—C5—Fe1	69.78 (12)	H27A—C27—H27C	109.5
C1—C5—H5	126.2	H27B—C27—H27C	109.5
C4—C5—H5	126.2		

Table S4. Bond lengths (Å) and bond angles (°) for **6**.

C1—C2	1.403 (9)	C16—H16	0.9300
C1—C5	1.413 (8)	C17—H17	0.9300
C1—Fe1	2.033 (6)	C18—O2	1.427 (6)
C1—H1	0.9800	C18—H18A	0.9600
C2—C3	1.411 (10)	C18—H18B	0.9600
C2—Fe1	2.030 (6)	C18—H18C	0.9600
C2—H2	0.9800	C19—N1	1.474 (5)
C3—C4	1.400 (9)	C19—C20	1.510 (7)
C3—Fe1	2.038 (6)	C19—C26	1.544 (6)
C3—H3	0.9800	C19—H19	0.9800
C4—C5	1.405 (8)	C20—C21	1.387 (7)
C4—Fe1	2.040 (5)	C20—C25	1.396 (7)
C4—H4	0.9800	C21—C22	1.384 (7)
C5—Fe1	2.045 (5)	C21—H21	0.9300
C5—H5	0.9800	C22—C23	1.372 (8)
C6—C7	1.425 (7)	C22—H22	0.9300
C6—C10	1.445 (6)	C23—C24	1.380 (7)
C6—Fe1	2.048 (5)	C23—Cl1	1.754 (5)
C6—H6	0.9800	C24—C25	1.374 (7)
C7—C8	1.420 (7)	C24—H24	0.9300
C7—Fe1	2.044 (5)	C25—H25	0.9300
C7—H7	0.9800	C26—O3	1.228 (6)
C8—C9	1.409 (7)	C26—N2	1.335 (6)
C8—Fe1	2.053 (5)	C27—N2	1.479 (6)
C8—H8	0.9800	C27—C28	1.521 (7)
C9—C10	1.437 (6)	C27—C29	1.521 (8)
C9—Fe1	2.036 (5)	C27—C30	1.529 (7)
C9—H9	0.9800	C28—H28A	0.9600
C10—C11	1.478 (6)	C28—H28B	0.9600
C10—Fe1	2.032 (5)	C28—H28C	0.9600

C11—O1	1.235 (6)	C29—H29A	0.9600
C11—N1	1.365 (6)	C29—H29B	0.9600
C12—C13	1.387 (6)	C29—H29C	0.9600
C12—C17	1.391 (6)	C30—H30A	0.9600
C12—N1	1.434 (5)	C30—H30B	0.9600
C13—C14	1.376 (6)	C30—H30C	0.9600
C13—H13	0.9300	C31—Cl2	1.685 (9)
C14—C15	1.397 (7)	C31—Cl3	1.734 (8)
C14—H14	0.9300	C31—H31A	0.9700
C15—O2	1.362 (5)	C31—H31B	0.9700
C15—C16	1.378 (7)	N2—H2A	0.8600
C16—C17	1.393 (7)		
C2—C1—C5	108.4 (5)	C20—C21—H21	119.3
C2—C1—Fe1	69.7 (3)	C23—C22—C21	119.1 (5)
C5—C1—Fe1	70.2 (3)	C23—C22—H22	120.4
C2—C1—H1	125.8	C21—C22—H22	120.4
C5—C1—H1	125.8	C22—C23—C24	121.2 (5)
Fe1—C1—H1	125.8	C22—C23—Cl1	119.8 (4)
C1—C2—C3	107.8 (5)	C24—C23—Cl1	119.1 (4)
C1—C2—Fe1	69.9 (3)	C25—C24—C23	119.1 (5)
C3—C2—Fe1	70.0 (3)	C25—C24—H24	120.4
C1—C2—H2	126.1	C23—C24—H24	120.4
C3—C2—H2	126.1	C24—C25—C20	121.4 (5)
Fe1—C2—H2	126.1	C24—C25—H25	119.3
C4—C3—C2	107.9 (5)	C20—C25—H25	119.3
C4—C3—Fe1	70.0 (3)	O3—C26—N2	124.8 (4)
C2—C3—Fe1	69.4 (4)	O3—C26—C19	122.0 (4)
C4—C3—H3	126.0	N2—C26—C19	113.1 (4)
C2—C3—H3	126.0	N2—C27—C28	106.5 (4)
Fe1—C3—H3	126.0	N2—C27—C29	110.5 (4)
C3—C4—C5	108.6 (6)	C28—C27—C29	110.1 (4)
C3—C4—Fe1	69.9 (3)	N2—C27—C30	110.4 (4)
C5—C4—Fe1	70.1 (3)	C28—C27—C30	109.0 (4)
C3—C4—H4	125.7	C29—C27—C30	110.2 (5)
C5—C4—H4	125.7	C27—C28—H28A	109.5
Fe1—C4—H4	125.7	C27—C28—H28B	109.5
C4—C5—C1	107.3 (5)	H28A—C28—H28B	109.5
C4—C5—Fe1	69.7 (3)	C27—C28—H28C	109.5
C1—C5—Fe1	69.3 (3)	H28A—C28—H28C	109.5
C4—C5—H5	126.3	H28B—C28—H28C	109.5
C1—C5—H5	126.3	C27—C29—H29A	109.5
Fe1—C5—H5	126.3	C27—C29—H29B	109.5
C7—C6—C10	107.1 (4)	H29A—C29—H29B	109.5
C7—C6—Fe1	69.5 (3)	C27—C29—H29C	109.5
C10—C6—Fe1	68.7 (3)	H29A—C29—H29C	109.5
C7—C6—H6	126.4	H29B—C29—H29C	109.5
C10—C6—H6	126.4	C27—C30—H30A	109.5
Fe1—C6—H6	126.4	C27—C30—H30B	109.5
C8—C7—C6	109.1 (4)	H30A—C30—H30B	109.5
C8—C7—Fe1	70.0 (3)	C27—C30—H30C	109.5
C6—C7—Fe1	69.8 (3)	H30A—C30—H30C	109.5
C8—C7—H7	125.4	H30B—C30—H30C	109.5
C6—C7—H7	125.4	Cl2—C31—Cl3	114.6 (4)
Fe1—C7—H7	125.4	Cl2—C31—H31A	108.6
C9—C8—C7	107.8 (4)	Cl3—C31—H31A	108.6
C9—C8—Fe1	69.2 (3)	Cl2—C31—H31B	108.6
C7—C8—Fe1	69.4 (3)	Cl3—C31—H31B	108.6
C9—C8—H8	126.1	H31A—C31—H31B	107.6

C7—C8—H8	126.1	C2—Fe1—C10	164.7 (3)
Fe1—C8—H8	126.1	C2—Fe1—C1	40.4 (3)
C8—C9—C10	108.9 (4)	C10—Fe1—C1	127.1 (2)
C8—C9—Fe1	70.5 (3)	C2—Fe1—C9	152.6 (2)
C10—C9—Fe1	69.2 (3)	C10—Fe1—C9	41.38 (18)
C8—C9—H9	125.5	C1—Fe1—C9	165.8 (2)
C10—C9—H9	125.5	C2—Fe1—C3	40.6 (3)
Fe1—C9—H9	125.5	C10—Fe1—C3	153.2 (3)
C9—C10—C6	107.0 (4)	C1—Fe1—C3	67.9 (3)
C9—C10—C11	118.6 (4)	C9—Fe1—C3	119.0 (3)
C6—C10—C11	134.3 (4)	C2—Fe1—C4	67.9 (3)
C9—C10—Fe1	69.4 (3)	C10—Fe1—C4	119.3 (2)
C6—C10—Fe1	69.9 (3)	C1—Fe1—C4	67.7 (2)
C11—C10—Fe1	123.6 (3)	C9—Fe1—C4	108.7 (2)
O1—C11—N1	119.7 (4)	C3—Fe1—C4	40.2 (3)
O1—C11—C10	118.7 (4)	C2—Fe1—C7	107.6 (2)
N1—C11—C10	121.6 (4)	C10—Fe1—C7	69.01 (18)
C13—C12—C17	119.9 (4)	C1—Fe1—C7	119.0 (2)
C13—C12—N1	120.6 (4)	C9—Fe1—C7	68.13 (19)
C17—C12—N1	119.4 (4)	C3—Fe1—C7	127.0 (2)
C14—C13—C12	119.6 (4)	C4—Fe1—C7	164.7 (2)
C14—C13—H13	120.2	C2—Fe1—C5	68.2 (2)
C12—C13—H13	120.2	C10—Fe1—C5	107.8 (2)
C13—C14—C15	120.6 (4)	C1—Fe1—C5	40.5 (2)
C13—C14—H14	119.7	C9—Fe1—C5	127.9 (2)
C15—C14—H14	119.7	C3—Fe1—C5	67.8 (2)
O2—C15—C16	124.3 (4)	C4—Fe1—C5	40.3 (2)
O2—C15—C14	115.7 (4)	C7—Fe1—C5	153.3 (2)
C16—C15—C14	119.9 (4)	C2—Fe1—C6	126.3 (2)
C15—C16—C17	119.6 (4)	C10—Fe1—C6	41.49 (18)
C15—C16—H16	120.2	C1—Fe1—C6	107.6 (2)
C17—C16—H16	120.2	C9—Fe1—C6	69.13 (18)
C12—C17—C16	120.2 (4)	C3—Fe1—C6	164.3 (3)
C12—C17—H17	119.9	C4—Fe1—C6	153.7 (2)
C16—C17—H17	119.9	C7—Fe1—C6	40.74 (18)
O2—C18—H18A	109.5	C5—Fe1—C6	119.2 (2)
O2—C18—H18B	109.5	C2—Fe1—C8	118.7 (2)
H18A—C18—H18B	109.5	C10—Fe1—C8	69.09 (18)
O2—C18—H18C	109.5	C1—Fe1—C8	152.8 (2)
H18A—C18—H18C	109.5	C9—Fe1—C8	40.31 (19)
H18B—C18—H18C	109.5	C3—Fe1—C8	107.9 (2)
N1—C19—C20	112.3 (4)	C4—Fe1—C8	127.5 (2)
N1—C19—C26	109.9 (4)	C7—Fe1—C8	40.56 (18)
C20—C19—C26	110.8 (4)	C5—Fe1—C8	165.1 (2)
N1—C19—H19	107.9	C6—Fe1—C8	68.83 (18)
C20—C19—H19	107.9	C11—N1—C12	123.0 (4)
C26—C19—H19	107.9	C11—N1—C19	115.7 (4)
C21—C20—C25	117.8 (5)	C12—N1—C19	120.8 (4)
C21—C20—C19	122.6 (4)	C26—N2—C27	125.7 (4)
C25—C20—C19	119.6 (4)	C26—N2—H2A	117.1
C22—C21—C20	121.4 (5)	C27—N2—H2A	117.1
C22—C21—H21	119.3	C15—O2—C18	117.5 (4)

Table S5. Bond lengths (Å) and bond angles (°) for **9**.

Fe2—C22	2.036 (3)	C5—H5	0.9300
Fe2—C23	2.041 (2)	C23—C24	1.414 (4)
Fe2—C27	2.041 (3)	C23—C22	1.424 (4)

Fe2—C28	2.042 (3)	C23—H23	0.9300
Fe2—C26	2.042 (3)	C21—C22	1.412 (4)
Fe2—C21	2.043 (3)	C21—C20	1.423 (3)
Fe2—C24	2.044 (2)	C21—H21	0.9300
Fe2—C25	2.046 (3)	C15—C18	1.502 (4)
Fe2—C29	2.048 (3)	C29—C25	1.413 (4)
Fe2—C20	2.049 (2)	C29—C28	1.414 (4)
Fe1—C9	2.032 (2)	C29—H29	0.9300
Fe1—C3	2.039 (3)	N1—C11	1.363 (3)
Fe1—C2	2.039 (3)	N1—C19	1.496 (3)
Fe1—C10	2.041 (2)	C11—C10	1.492 (3)
Fe1—C1	2.044 (3)	C20—C24	1.433 (3)
Fe1—C6	2.046 (3)	C20—C19	1.504 (3)
Fe1—C4	2.049 (3)	C19—H19	0.9800
Fe1—C8	2.049 (3)	C10—C9	1.436 (4)
Fe1—C7	2.050 (3)	C10—C6	1.438 (4)
Fe1—C5	2.052 (3)	C9—H9	0.9300
O2—C30	1.231 (3)	C1—C2	1.419 (4)
C31—N2	1.479 (3)	C1—H1	0.9300
C31—C33	1.527 (3)	C24—H24	0.9300
C31—C32	1.529 (4)	C26—C27	1.413 (4)
C31—C34	1.533 (4)	C26—C25	1.420 (4)
O1—C11	1.240 (3)	C26—H26	0.9300
C17—C12	1.376 (4)	C22—H22	0.9300
C17—C16	1.396 (4)	C7—C6	1.410 (4)
C17—H17	0.9300	C7—H7	0.9300
C34—H34A	0.9600	C32—H32A	0.9600
C34—H34B	0.9600	C32—H32B	0.9600
C34—H34C	0.9600	C32—H32C	0.9600
C8—C7	1.415 (4)	C6—H6	0.9300
C8—C9	1.420 (3)	C27—C28	1.417 (4)
C8—H8	0.9300	C27—H27	0.9300
N2—C30	1.335 (3)	C2—C3	1.402 (4)
N2—H2	0.8600	C2—H2A	0.9300
C13—C14	1.383 (4)	C33—H33A	0.9600
C13—C12	1.391 (3)	C33—H33B	0.9600
C13—H13	0.9300	C33—H33C	0.9600
C16—C15	1.398 (4)	C28—H28	0.9300
C16—H16	0.9300	C18—H18A	0.9600
C14—C15	1.394 (4)	C18—H18B	0.9600
C14—H14	0.9300	C18—H18C	0.9600
C30—C19	1.550 (3)	C25—H25	0.9300
C12—N1	1.442 (3)	C3—C4	1.420 (4)
C5—C4	1.415 (4)	C3—H3	0.9300
C5—C1	1.416 (4)	C4—H4	0.9300
C22—Fe2—C23	40.89 (10)	C24—C23—H23	126.1
C22—Fe2—C27	117.71 (11)	C22—C23—H23	126.1
C23—Fe2—C27	106.81 (11)	Fe2—C23—H23	126.2
C22—Fe2—C28	105.26 (11)	C22—C21—C20	108.5 (2)
C23—Fe2—C28	124.52 (11)	C22—C21—Fe2	69.50 (15)
C27—Fe2—C28	40.61 (11)	C20—C21—Fe2	69.88 (14)
C22—Fe2—C26	153.16 (11)	C22—C21—H21	125.7
C23—Fe2—C26	119.96 (11)	C20—C21—H21	125.7
C27—Fe2—C26	40.49 (11)	Fe2—C21—H21	126.5
C28—Fe2—C26	68.29 (12)	C14—C15—C16	118.2 (2)
C22—Fe2—C21	40.51 (10)	C14—C15—C18	120.6 (2)
C23—Fe2—C21	68.47 (10)	C16—C15—C18	121.2 (3)
C27—Fe2—C21	151.96 (11)	C25—C29—C28	108.4 (2)

C28—Fe2—C21	117.79 (11)	C25—C29—Fe2	69.72 (15)
C26—Fe2—C21	165.80 (11)	C28—C29—Fe2	69.53 (15)
C22—Fe2—C24	68.41 (10)	C25—C29—H29	125.8
C23—Fe2—C24	40.52 (10)	C28—C29—H29	125.8
C27—Fe2—C24	126.92 (11)	Fe2—C29—H29	126.5
C28—Fe2—C24	162.91 (11)	C11—N1—C12	122.9 (2)
C26—Fe2—C24	109.75 (11)	C11—N1—C19	116.2 (2)
C21—Fe2—C24	68.42 (10)	C12—N1—C19	120.86 (19)
C22—Fe2—C25	163.15 (11)	O1—C11—N1	119.7 (2)
C23—Fe2—C25	155.52 (11)	O1—C11—C10	118.8 (2)
C27—Fe2—C25	68.18 (11)	N1—C11—C10	121.4 (2)
C28—Fe2—C25	68.24 (12)	C21—C20—C24	107.1 (2)
C26—Fe2—C25	40.66 (10)	C21—C20—C19	127.4 (2)
C21—Fe2—C25	127.40 (10)	C24—C20—C19	125.2 (2)
C24—Fe2—C25	122.15 (11)	C21—C20—Fe2	69.41 (14)
C22—Fe2—C29	124.82 (11)	C24—C20—Fe2	69.31 (13)
C23—Fe2—C29	162.02 (12)	C19—C20—Fe2	130.89 (17)
C27—Fe2—C29	68.00 (11)	N1—C19—C20	110.82 (19)
C28—Fe2—C29	40.46 (12)	N1—C19—C30	107.37 (19)
C26—Fe2—C29	68.06 (11)	C20—C19—C30	111.8 (2)
C21—Fe2—C29	107.53 (11)	N1—C19—H19	108.9
C24—Fe2—C29	156.02 (11)	C20—C19—H19	108.9
C25—Fe2—C29	40.39 (11)	C30—C19—H19	108.9
C22—Fe2—C20	68.59 (10)	C9—C10—C6	106.8 (2)
C23—Fe2—C20	68.75 (10)	C9—C10—C11	135.0 (2)
C27—Fe2—C20	165.54 (11)	C6—C10—C11	118.2 (2)
C28—Fe2—C20	153.25 (11)	C9—C10—Fe1	69.04 (14)
C26—Fe2—C20	128.73 (11)	C6—C10—Fe1	69.61 (14)
C21—Fe2—C20	40.71 (10)	C11—C10—Fe1	123.61 (16)
C24—Fe2—C20	40.99 (9)	C8—C9—C10	108.2 (2)
C25—Fe2—C20	109.79 (11)	C8—C9—Fe1	70.28 (14)
C29—Fe2—C20	120.44 (11)	C10—C9—Fe1	69.68 (14)
C9—Fe1—C3	158.81 (12)	C8—C9—H9	125.9
C9—Fe1—C2	122.81 (11)	C10—C9—H9	125.9
C3—Fe1—C2	40.22 (12)	Fe1—C9—H9	125.7
C9—Fe1—C10	41.28 (10)	C5—C1—C2	108.2 (3)
C3—Fe1—C10	158.65 (12)	C5—C1—Fe1	70.07 (16)
C2—Fe1—C10	159.59 (11)	C2—C1—Fe1	69.48 (15)
C9—Fe1—C1	107.41 (11)	C5—C1—H1	125.9
C3—Fe1—C1	67.94 (11)	C2—C1—H1	125.9
C2—Fe1—C1	40.67 (11)	Fe1—C1—H1	126.1
C10—Fe1—C1	123.28 (10)	C23—C24—C20	108.4 (2)
C9—Fe1—C6	68.88 (11)	C23—C24—Fe2	69.62 (14)
C3—Fe1—C6	122.55 (11)	C20—C24—Fe2	69.70 (13)
C2—Fe1—C6	157.66 (11)	C23—C24—H24	125.8
C10—Fe1—C6	41.21 (10)	C20—C24—H24	125.8
C1—Fe1—C6	160.30 (11)	Fe2—C24—H24	126.4
C9—Fe1—C4	158.75 (11)	C27—C26—C25	107.9 (3)
C3—Fe1—C4	40.65 (12)	C27—C26—Fe2	69.74 (16)
C2—Fe1—C4	68.08 (12)	C25—C26—Fe2	69.82 (15)
C10—Fe1—C4	122.57 (11)	C27—C26—H26	126.0
C1—Fe1—C4	67.90 (12)	C25—C26—H26	126.0
C6—Fe1—C4	108.14 (12)	Fe2—C26—H26	126.0
C9—Fe1—C8	40.73 (10)	C21—C22—C23	108.2 (2)
C3—Fe1—C8	122.90 (11)	C21—C22—Fe2	69.99 (15)
C2—Fe1—C8	107.21 (11)	C23—C22—Fe2	69.72 (15)
C10—Fe1—C8	68.91 (10)	C21—C22—H22	125.9
C1—Fe1—C8	122.47 (12)	C23—C22—H22	125.9

C6—Fe1—C8	68.15 (11)	Fe2—C22—H22	126.0
C4—Fe1—C8	159.42 (12)	C6—C7—C8	108.7 (2)
C9—Fe1—C7	68.41 (10)	C6—C7—Fe1	69.75 (15)
C3—Fe1—C7	107.73 (11)	C8—C7—Fe1	69.79 (15)
C2—Fe1—C7	122.13 (11)	C6—C7—H7	125.7
C10—Fe1—C7	68.71 (10)	C8—C7—H7	125.7
C1—Fe1—C7	158.07 (12)	Fe1—C7—H7	126.4
C6—Fe1—C7	40.26 (11)	C31—C32—H32A	109.5
C4—Fe1—C7	123.78 (12)	C31—C32—H32B	109.5
C8—Fe1—C7	40.38 (11)	H32A—C32—H32B	109.5
C9—Fe1—C5	122.63 (11)	C31—C32—H32C	109.5
C3—Fe1—C5	68.17 (12)	H32A—C32—H32C	109.5
C2—Fe1—C5	68.30 (11)	H32B—C32—H32C	109.5
C10—Fe1—C5	107.54 (11)	C7—C6—C10	108.3 (2)
C1—Fe1—C5	40.46 (11)	C7—C6—Fe1	69.99 (15)
C6—Fe1—C5	124.06 (11)	C10—C6—Fe1	69.18 (14)
C4—Fe1—C5	40.39 (12)	C7—C6—H6	125.9
C8—Fe1—C5	158.45 (12)	C10—C6—H6	125.9
C7—Fe1—C5	159.94 (12)	Fe1—C6—H6	126.5
N2—C31—C33	109.6 (2)	C26—C27—C28	108.2 (2)
N2—C31—C32	110.2 (2)	C26—C27—Fe2	69.78 (15)
C33—C31—C32	111.1 (2)	C28—C27—Fe2	69.71 (16)
N2—C31—C34	106.6 (2)	C26—C27—H27	125.9
C33—C31—C34	109.8 (2)	C28—C27—H27	125.9
C32—C31—C34	109.4 (2)	Fe2—C27—H27	126.2
C12—C17—C16	120.0 (2)	C3—C2—C1	107.9 (3)
C12—C17—H17	120.0	C3—C2—Fe1	69.88 (16)
C16—C17—H17	120.0	C1—C2—Fe1	69.84 (15)
C31—C34—H34A	109.5	C3—C2—H2A	126.0
C31—C34—H34B	109.5	C1—C2—H2A	126.0
H34A—C34—H34B	109.5	Fe1—C2—H2A	125.8
C31—C34—H34C	109.5	C31—C33—H33A	109.5
H34A—C34—H34C	109.5	C31—C33—H33B	109.5
H34B—C34—H34C	109.5	H33A—C33—H33B	109.5
C7—C8—C9	108.1 (2)	C31—C33—H33C	109.5
C7—C8—Fe1	69.83 (15)	H33A—C33—H33C	109.5
C9—C8—Fe1	68.99 (14)	H33B—C33—H33C	109.5
C7—C8—H8	126.0	C29—C28—C27	107.7 (3)
C9—C8—H8	126.0	C29—C28—Fe2	70.01 (16)
Fe1—C8—H8	126.8	C27—C28—Fe2	69.68 (16)
C30—N2—C31	124.1 (2)	C29—C28—H28	126.1
C30—N2—H2	117.9	C27—C28—H28	126.1
C31—N2—H2	117.9	Fe2—C28—H28	125.8
C14—C13—C12	119.7 (2)	C15—C18—H18A	109.5
C14—C13—H13	120.1	C15—C18—H18B	109.5
C12—C13—H13	120.1	H18A—C18—H18B	109.5
C17—C16—C15	120.6 (2)	C15—C18—H18C	109.5
C17—C16—H16	119.7	H18A—C18—H18C	109.5
C15—C16—H16	119.7	H18B—C18—H18C	109.5
C13—C14—C15	121.3 (2)	C29—C25—C26	107.8 (3)
C13—C14—H14	119.4	C29—C25—Fe2	69.89 (16)
C15—C14—H14	119.4	C26—C25—Fe2	69.53 (15)
O2—C30—N2	125.2 (2)	C29—C25—H25	126.1
O2—C30—C19	120.0 (2)	C26—C25—H25	126.1
N2—C30—C19	114.9 (2)	Fe2—C25—H25	126.0
C17—C12—C13	120.2 (2)	C2—C3—C4	108.3 (3)
C17—C12—N1	121.0 (2)	C2—C3—Fe1	69.89 (15)
C13—C12—N1	118.8 (2)	C4—C3—Fe1	70.04 (15)

C4—C5—C1	107.6 (3)	C2—C3—H3	125.8
C4—C5—Fe1	69.70 (16)	C4—C3—H3	125.8
C1—C5—Fe1	69.48 (15)	Fe1—C3—H3	125.8
C4—C5—H5	126.2	C5—C4—C3	107.9 (3)
C1—C5—H5	126.2	C5—C4—Fe1	69.91 (15)
Fe1—C5—H5	126.2	C3—C4—Fe1	69.30 (16)
C24—C23—C22	107.8 (2)	C5—C4—H4	126.0
C24—C23—Fe2	69.86 (14)	C3—C4—H4	126.0
C22—C23—Fe2	69.39 (14)	Fe1—C4—H4	126.3

3. Cyclic voltammograms of compounds **2**-**8** and **10**.

Figure S1. Cyclic voltammogram of **2** (5×10^{-4} M) in CH_2Cl_2/CH_3CN (1:1, v/v) solution of n-Bu_4NPF_6 (0.1 M) at a scanning rate of 100 mV/s.

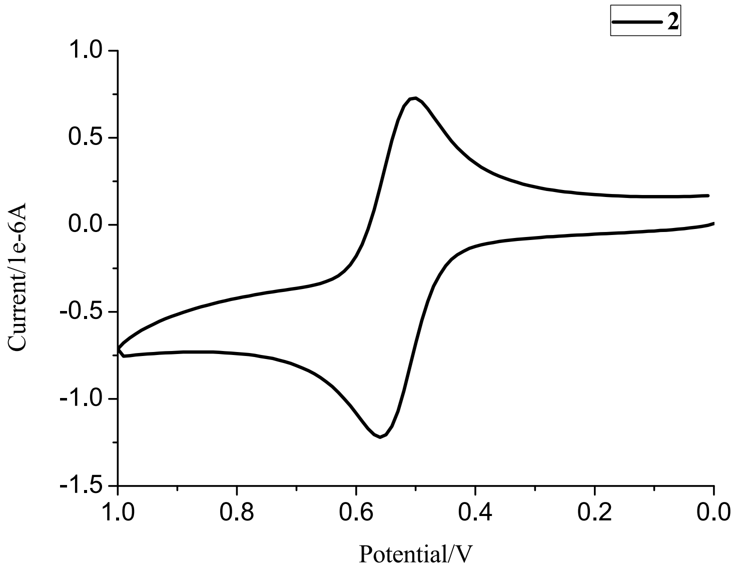

Figure S2. Cyclic voltammogram of **3** (5×10^{-4} M) in CH_2Cl_2/CH_3CN (1:1, v/v) solution of n-Bu_4NPF_6 (0.1 M) at a scanning rate of 100 mV/s.

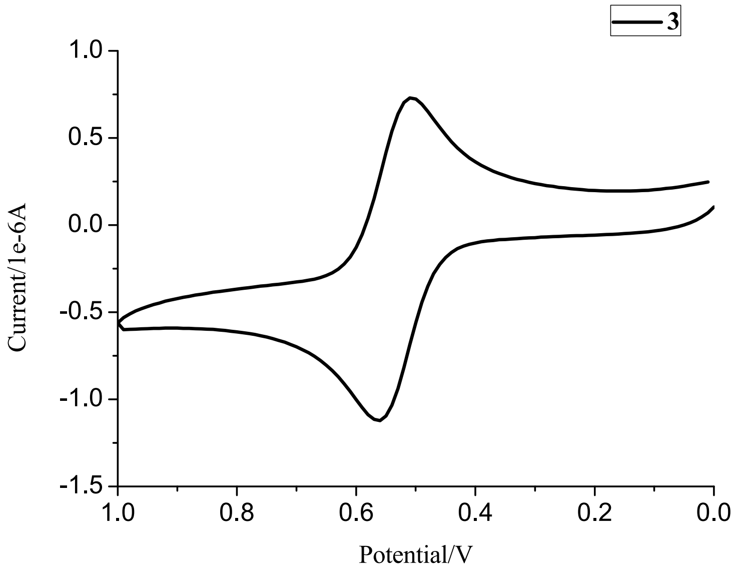

Figure S3. Cyclic voltammogram of **4** (5×10^{-4} M) in CH_2Cl_2/CH_3CN (1:1, v/v) solution of *n*-Bu_4NPF_6 (0.1 M) at a scanning rate of 100 mV/s.

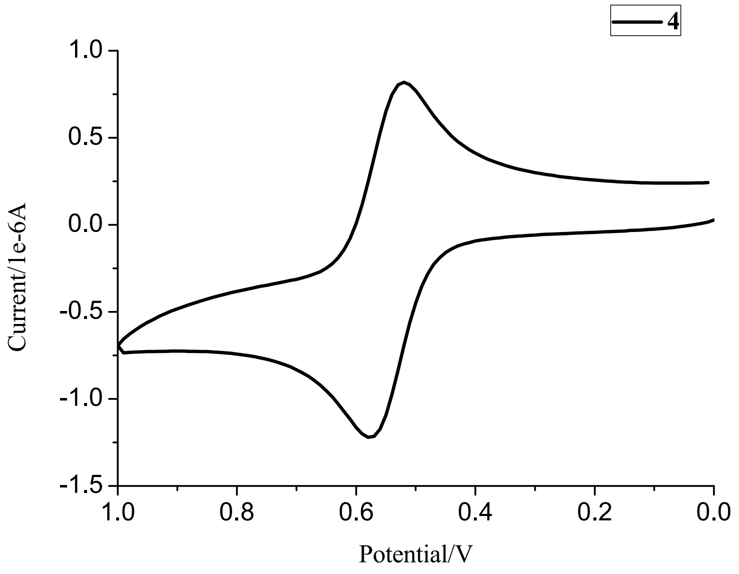

Figure S4. Cyclic voltammogram of **5** (5×10^{-4} M) in CH_2Cl_2/CH_3CN (1:1, v/v) solution of *n*-Bu_4NPF_6 (0.1 M) at a scanning rate of 100 mV/s.

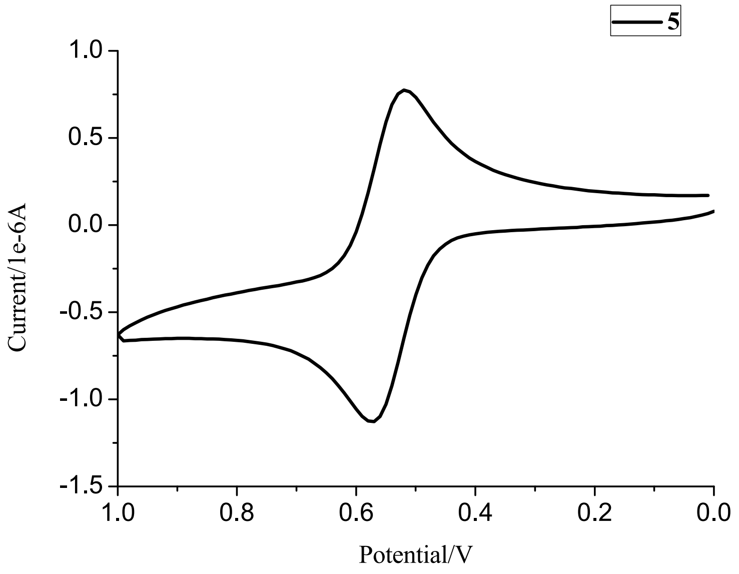

Figure S5. Cyclic voltammogram of **6** (5×10^{-4} M) in CH_2Cl_2/CH_3CN (1:1, v/v) solution of n-Bu_4NPF_6 (0.1 M) at a scanning rate of 100 mV/s.

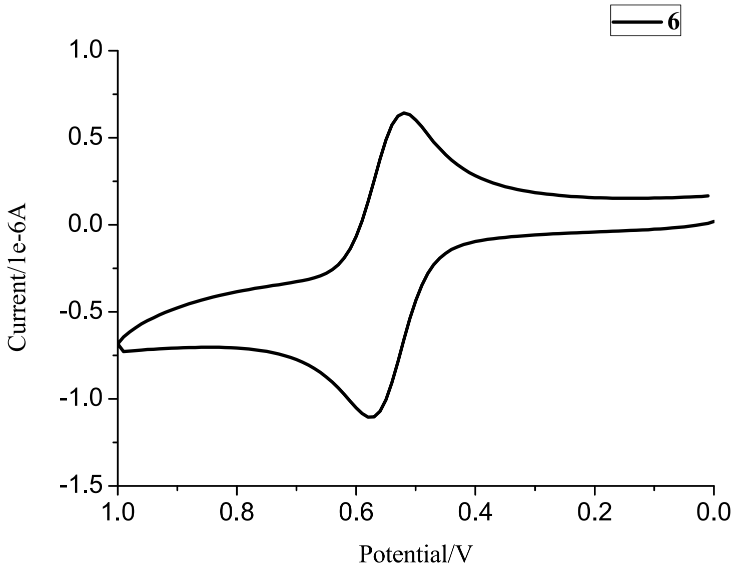

Figure S6. Cyclic voltammogram of **7** (5×10^{-4} M) in CH_2Cl_2/CH_3CN (1:1, v/v) solution of n-Bu_4NPF_6 (0.1 M) at a scanning rate of 100 mV/s.

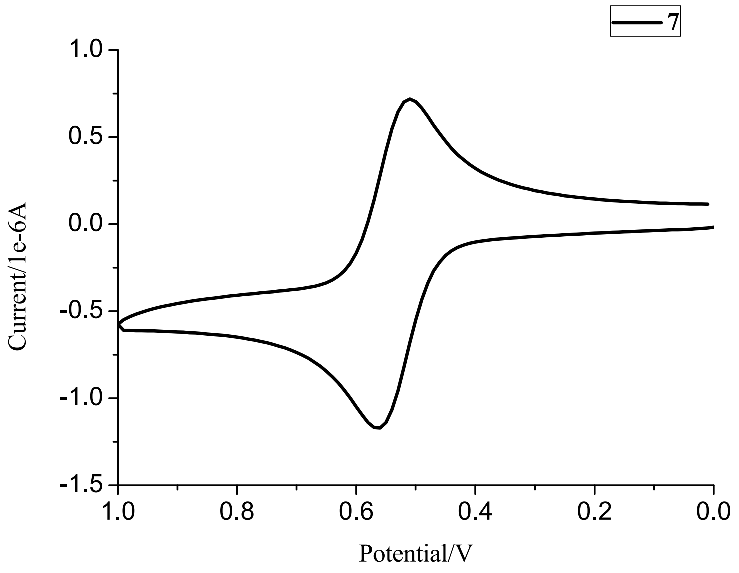

Figure S7. Cyclic voltammogram of **8** (5×10^{-4} M) in CH_2Cl_2/CH_3CN (1:1, v/v) solution of *n*-Bu_4NPF_6 (0.1 M) at a scanning rate of 100 mV/s.

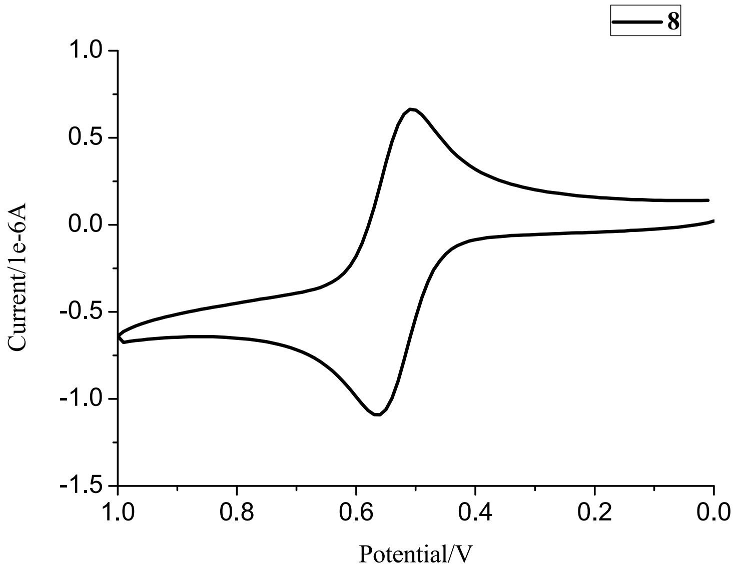

Figure S8. Cyclic voltammogram of **10** (5×10^{-4} M) in CH_2Cl_2/CH_3CN (1:1, v/v) solution of *n*-Bu_4NPF_6 (0.1 M) at a scanning rate of 100 mV/s.

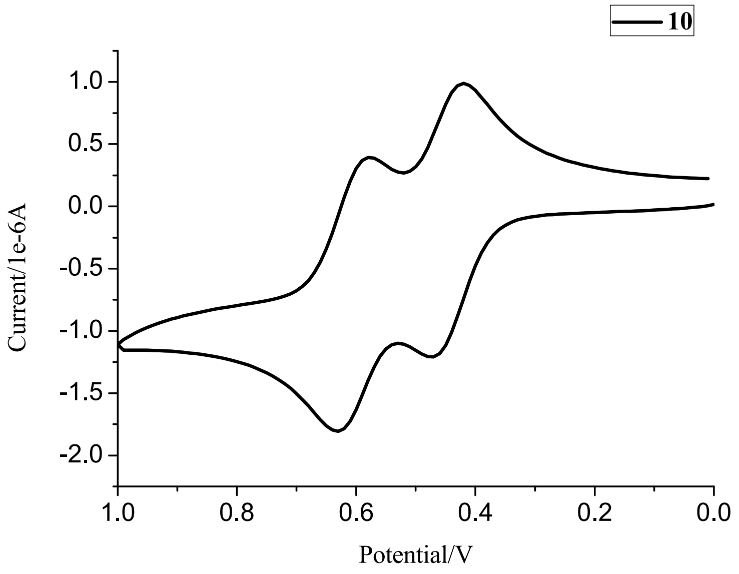

4. NMR of compounds **1-10**.

Figure S9. ^{1}H NMR of compound **1**

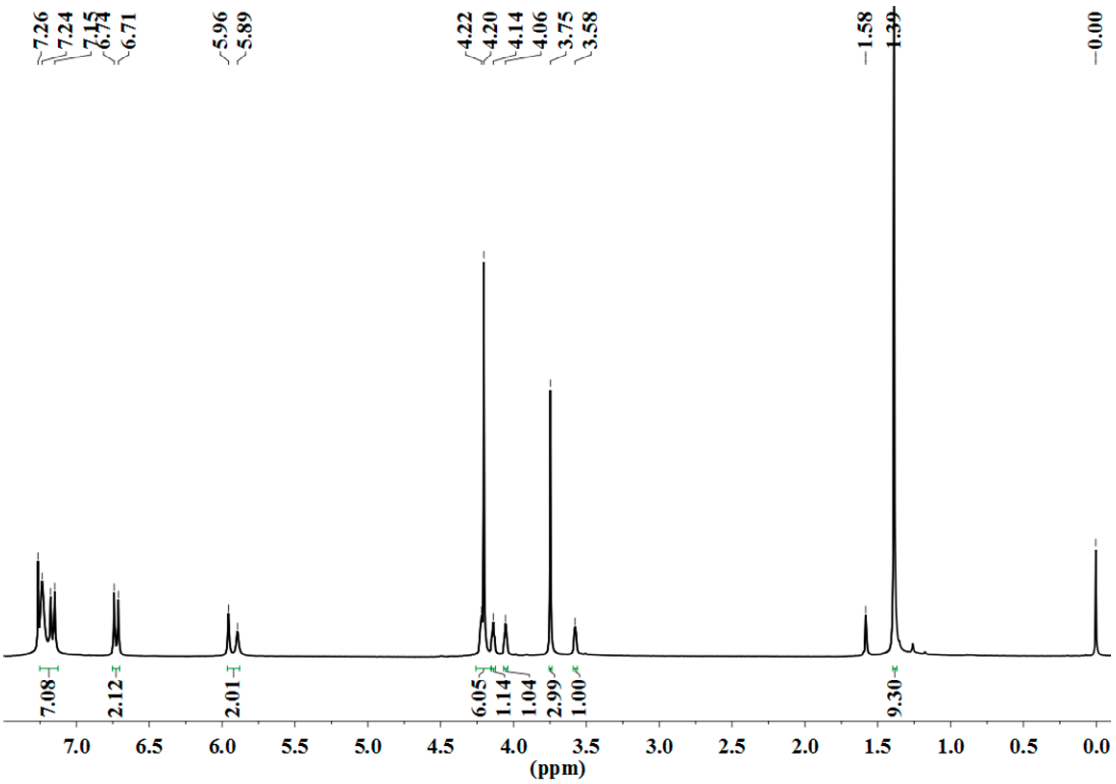

Figure S10. ^{13}C NMR of compound **1**

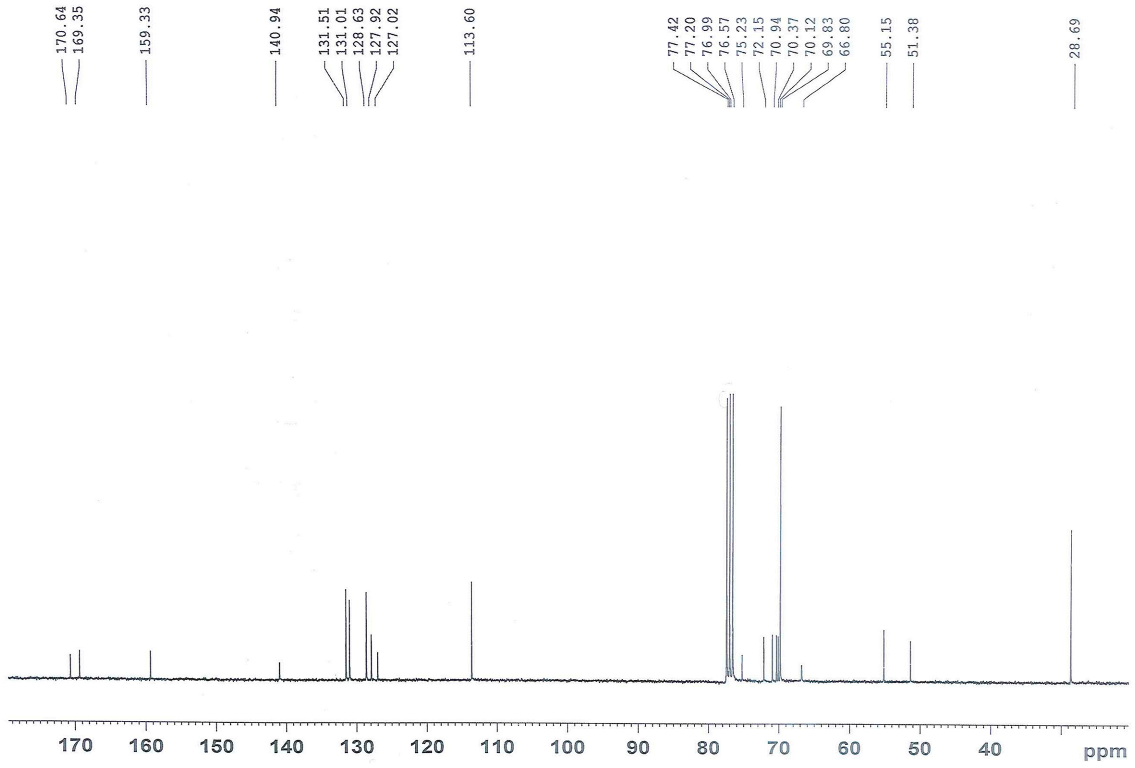

Figure S11. ^{1}H NMR of compound **2**

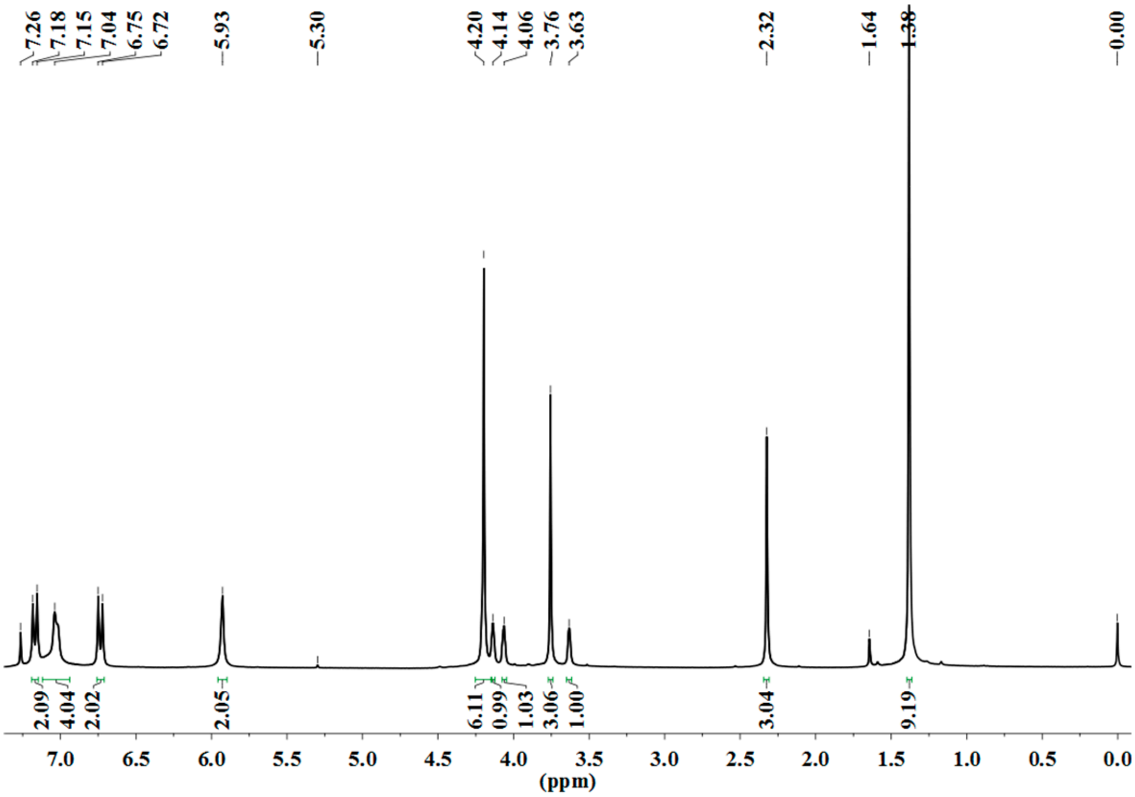

Figure S12. ^{13}C NMR of compound **2**

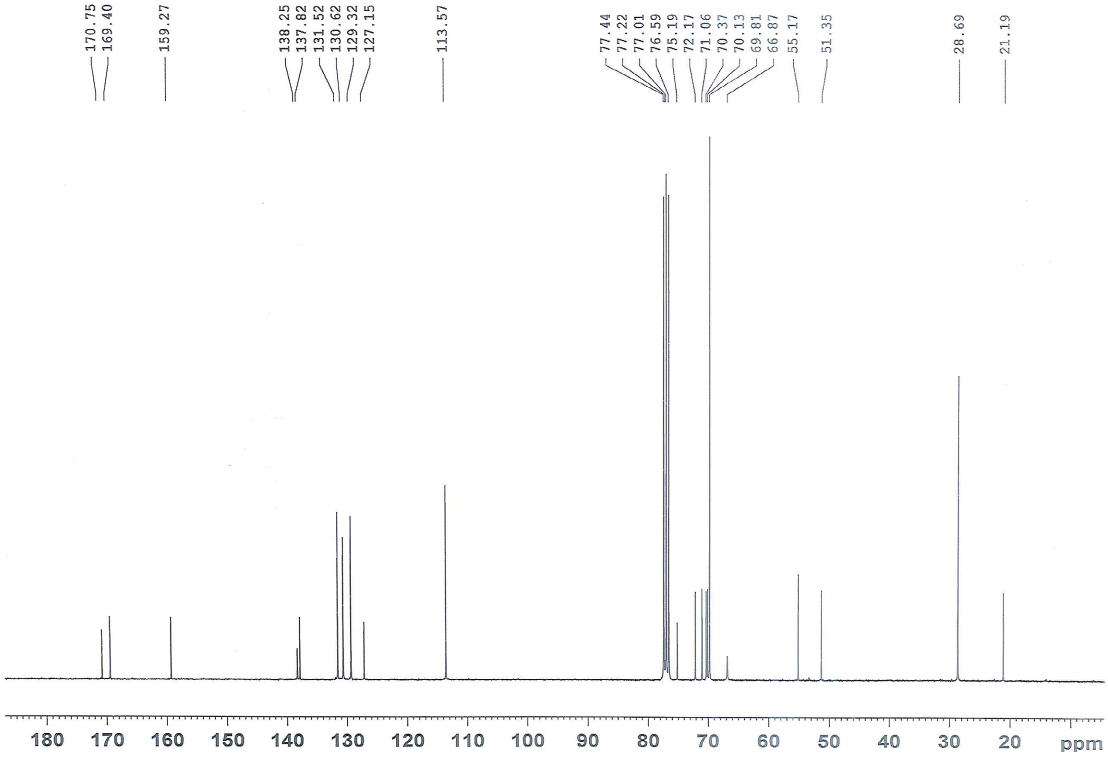

Figure S13. ^{1}H NMR of compound **3**

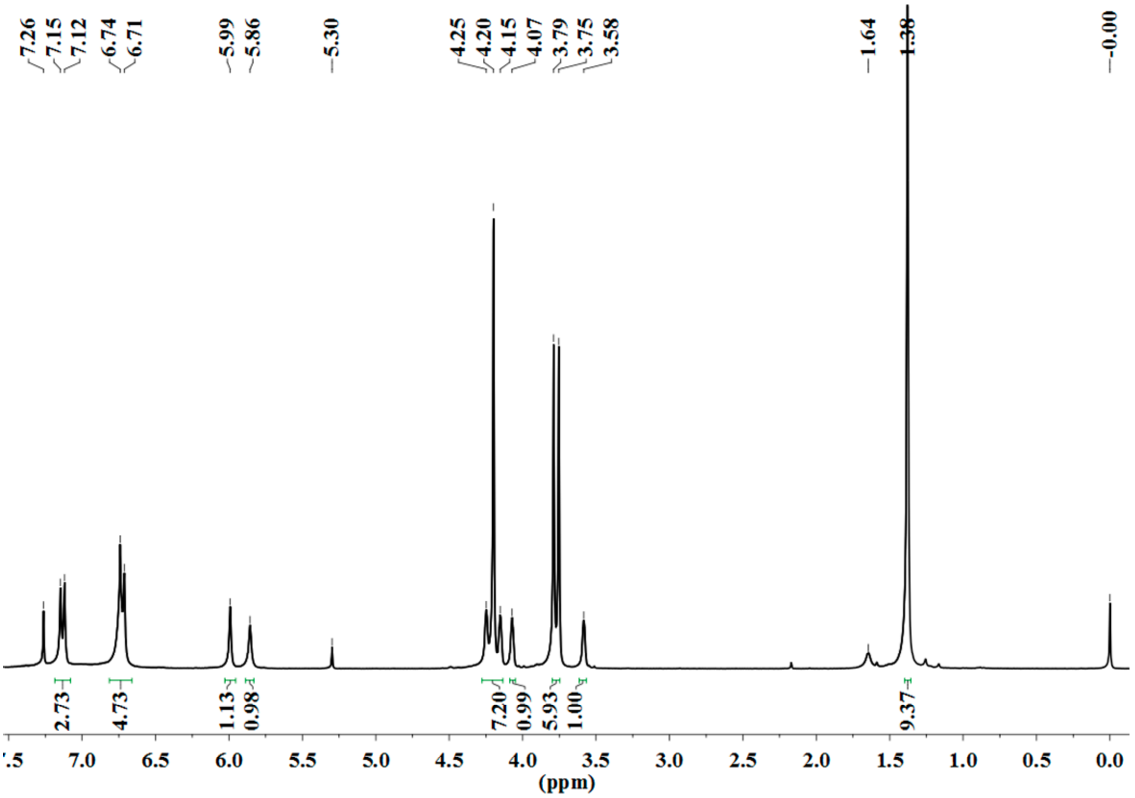

Figure S14. ^{13}C NMR of compound **3**

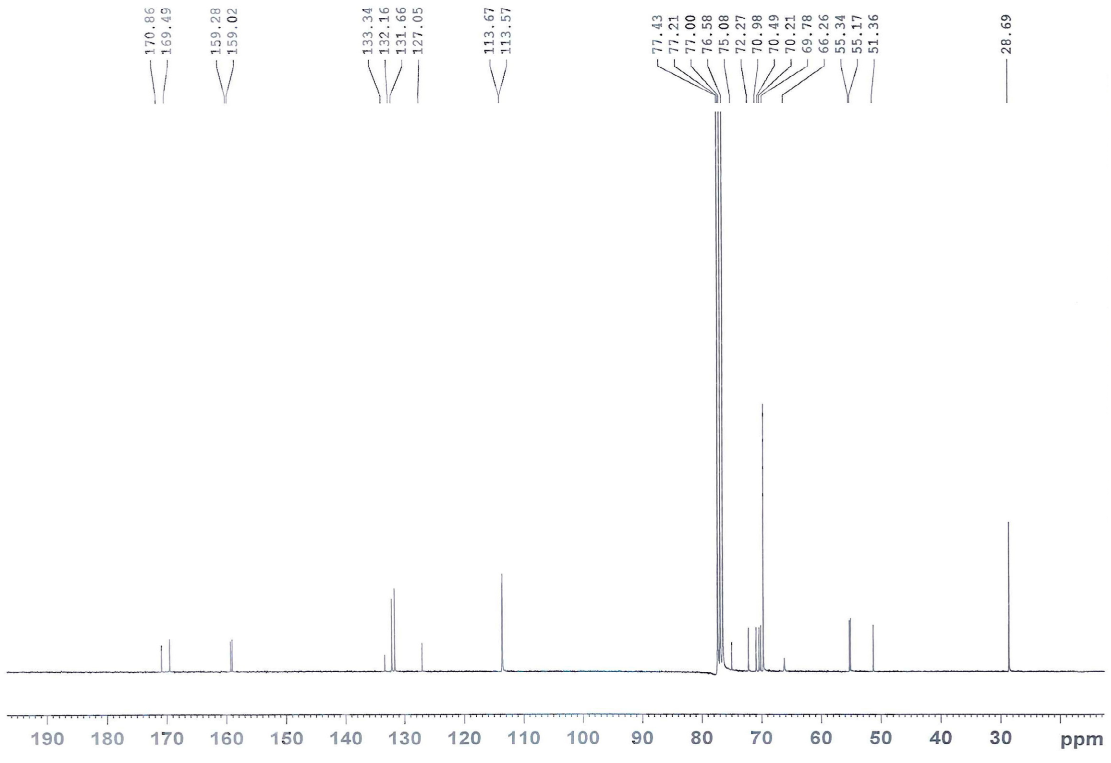

Figure S15. ^{1}H NMR of compound **4**

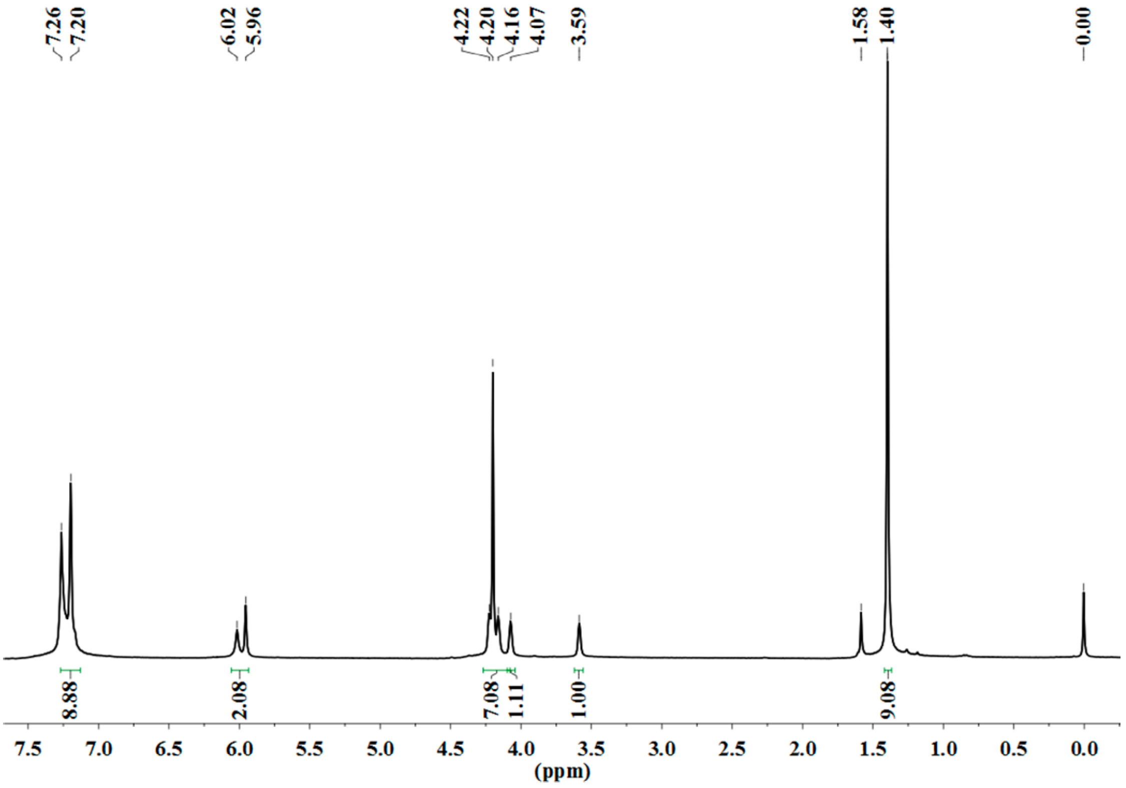

Figure S16. ^{13}C NMR of compound **4**

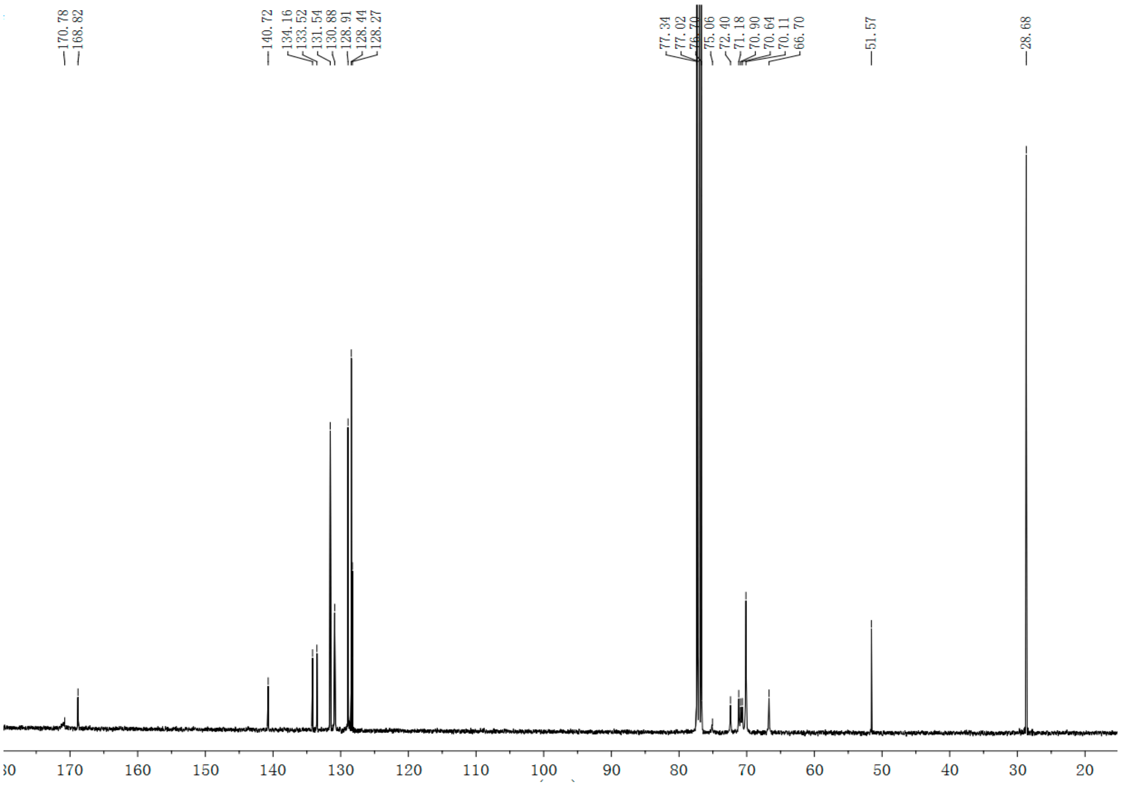

Figure S17. ^{1}H NMR of compound **5**

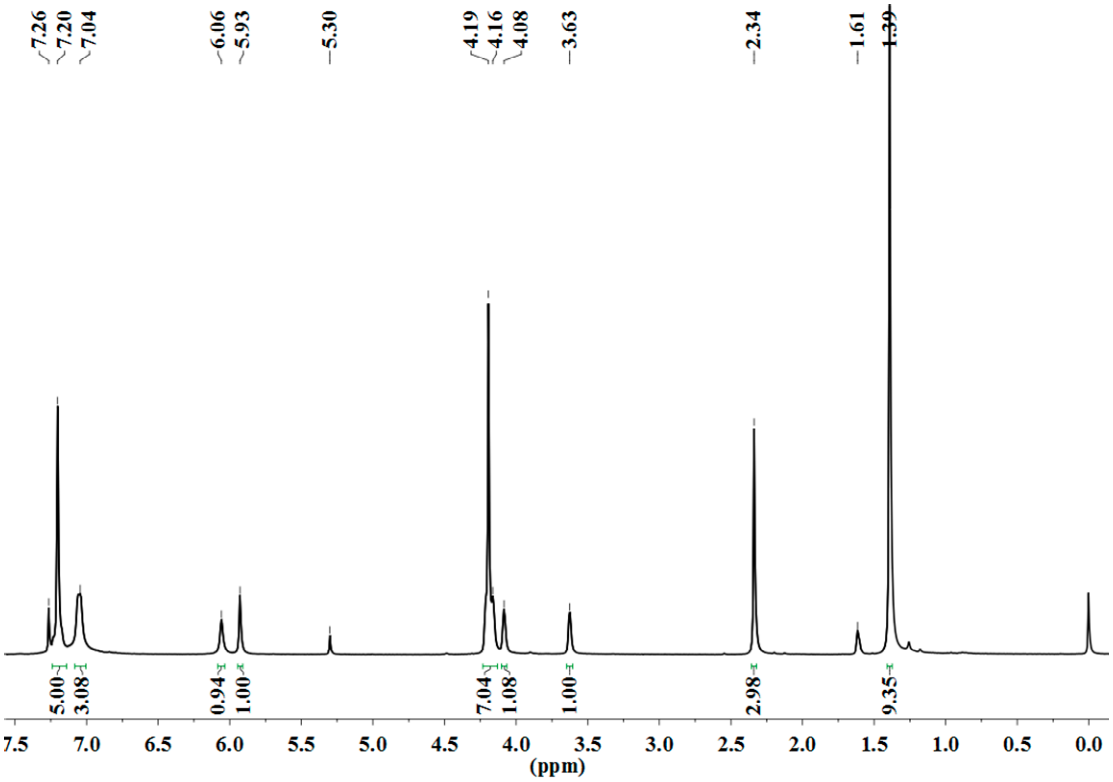

Figure S18. ^{13}C NMR of compound **5**

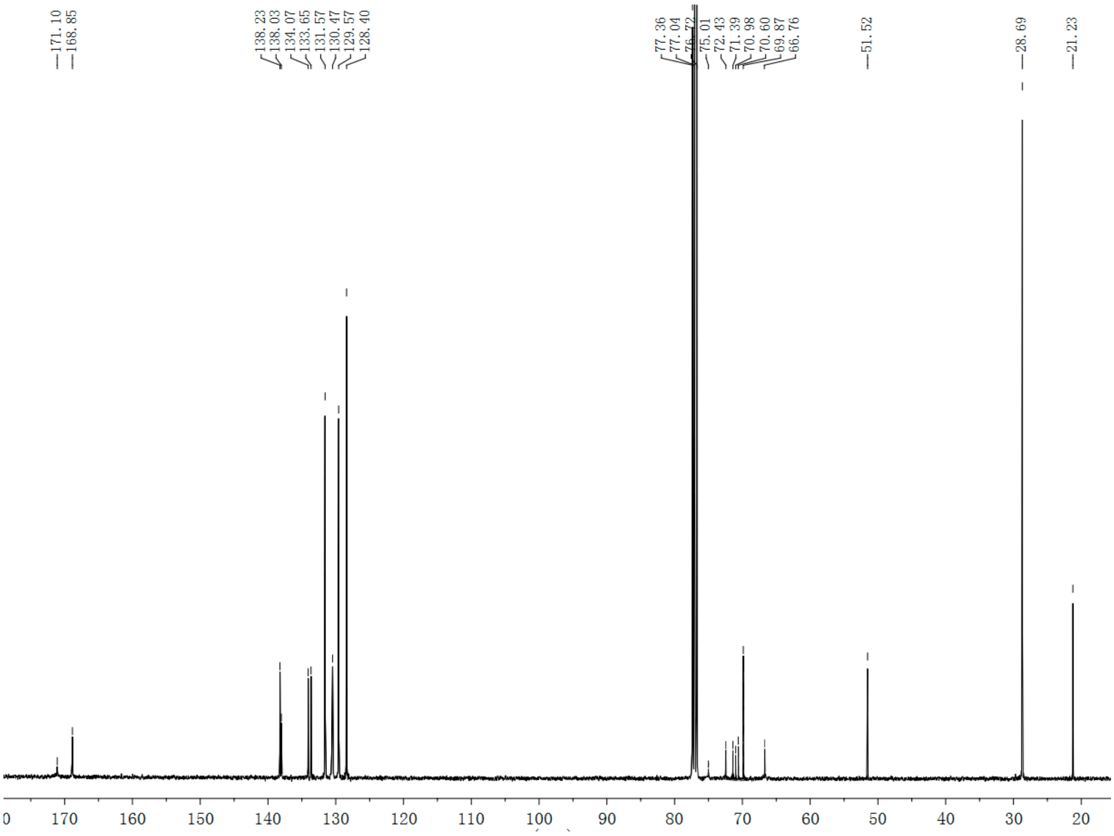

Figure S19. ^{1}H NMR of compound **6**

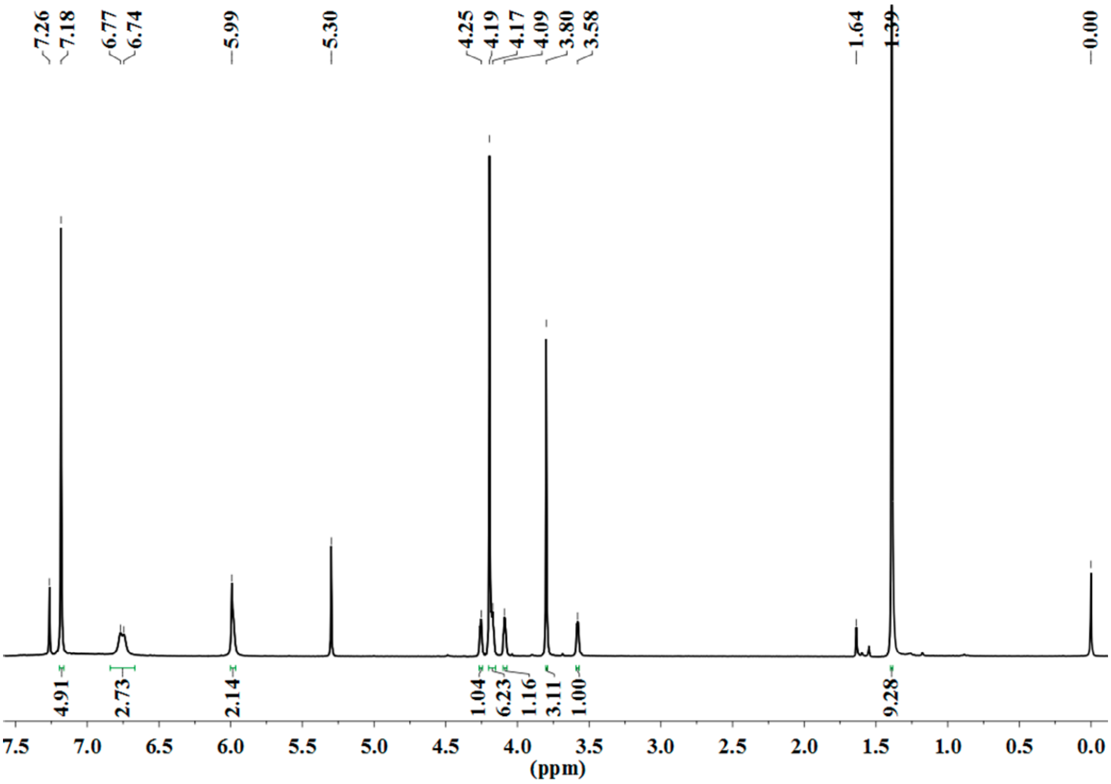

Figure S20. ^{13}C NMR of compound **6**

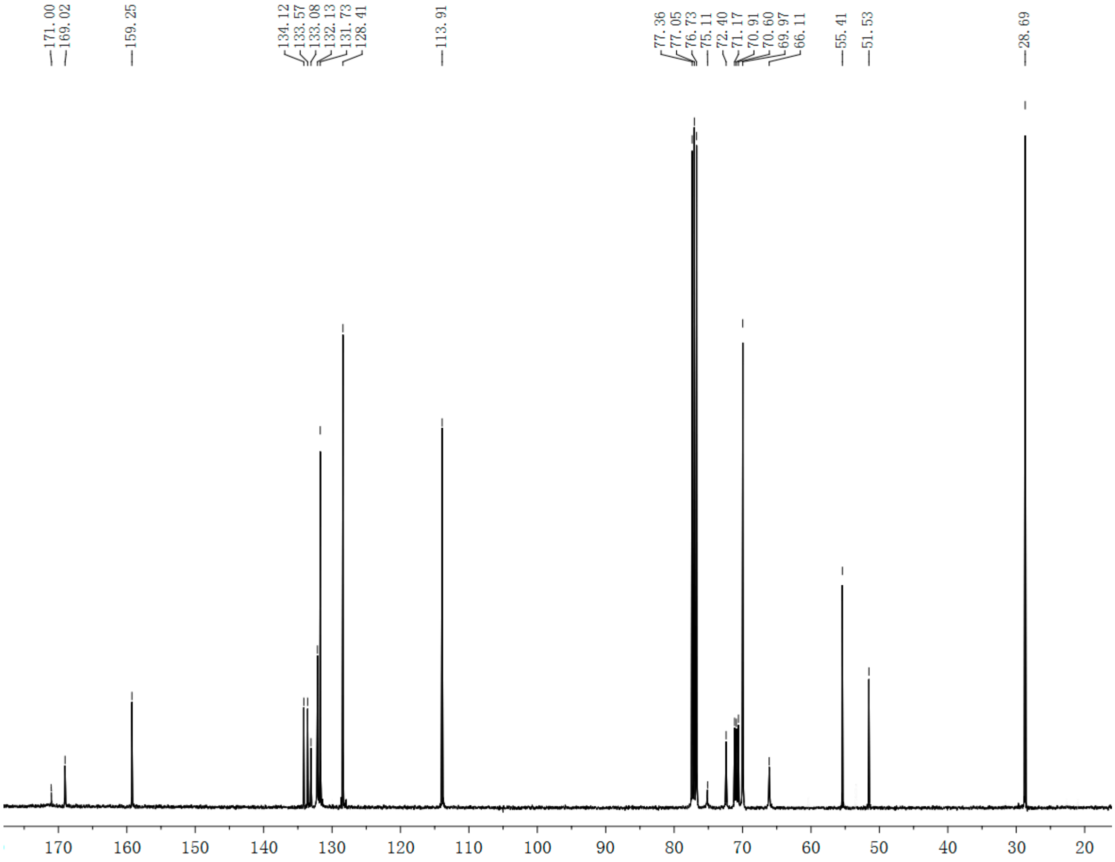

Fi

gure S21. ^{1}H NMR of compound **7**

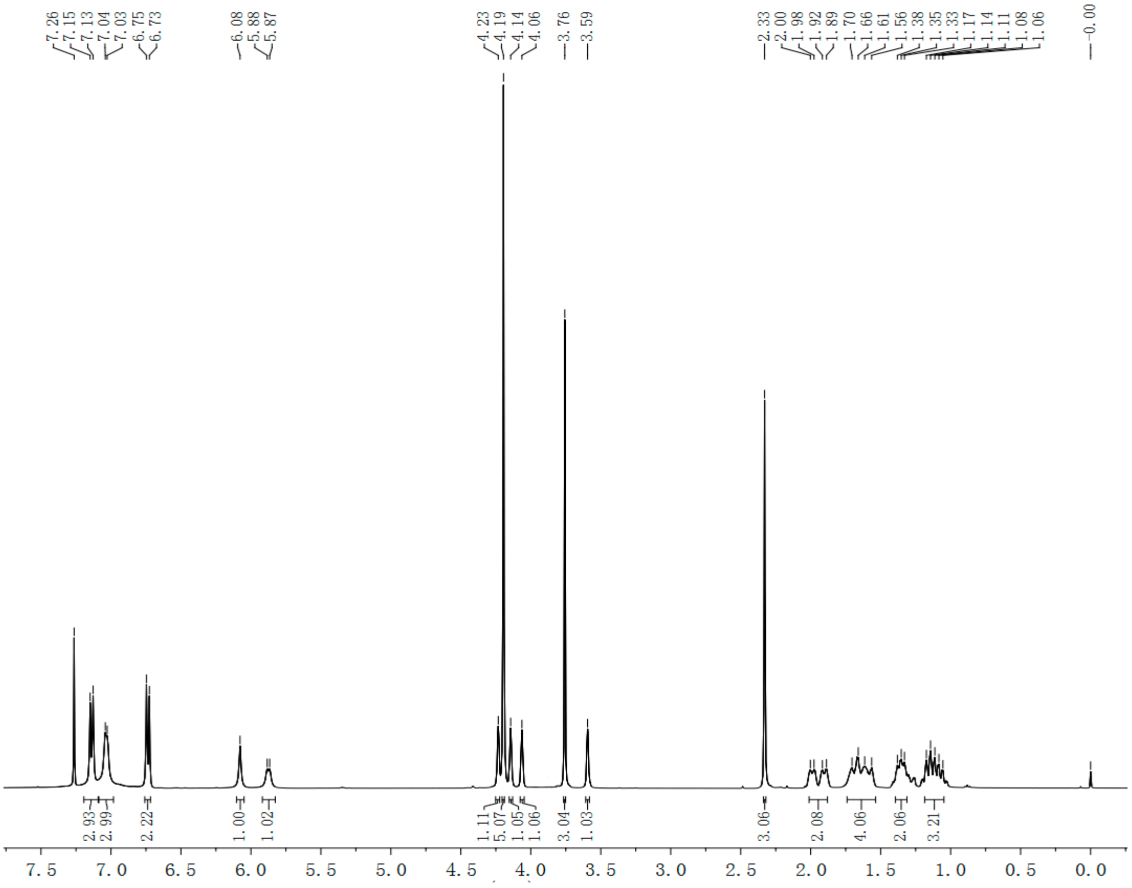

Figure S22. ^{13}C NMR of compound **7**

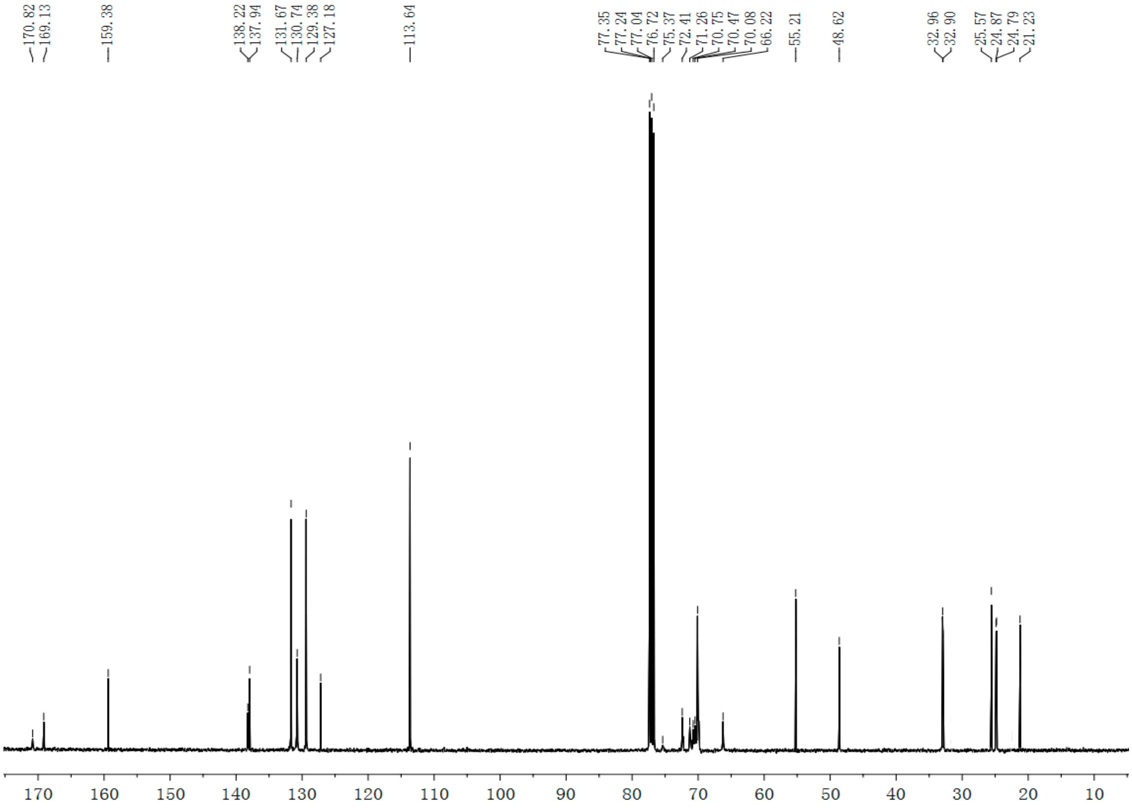

Figure S23. ^{1}H NMR of compound **8**

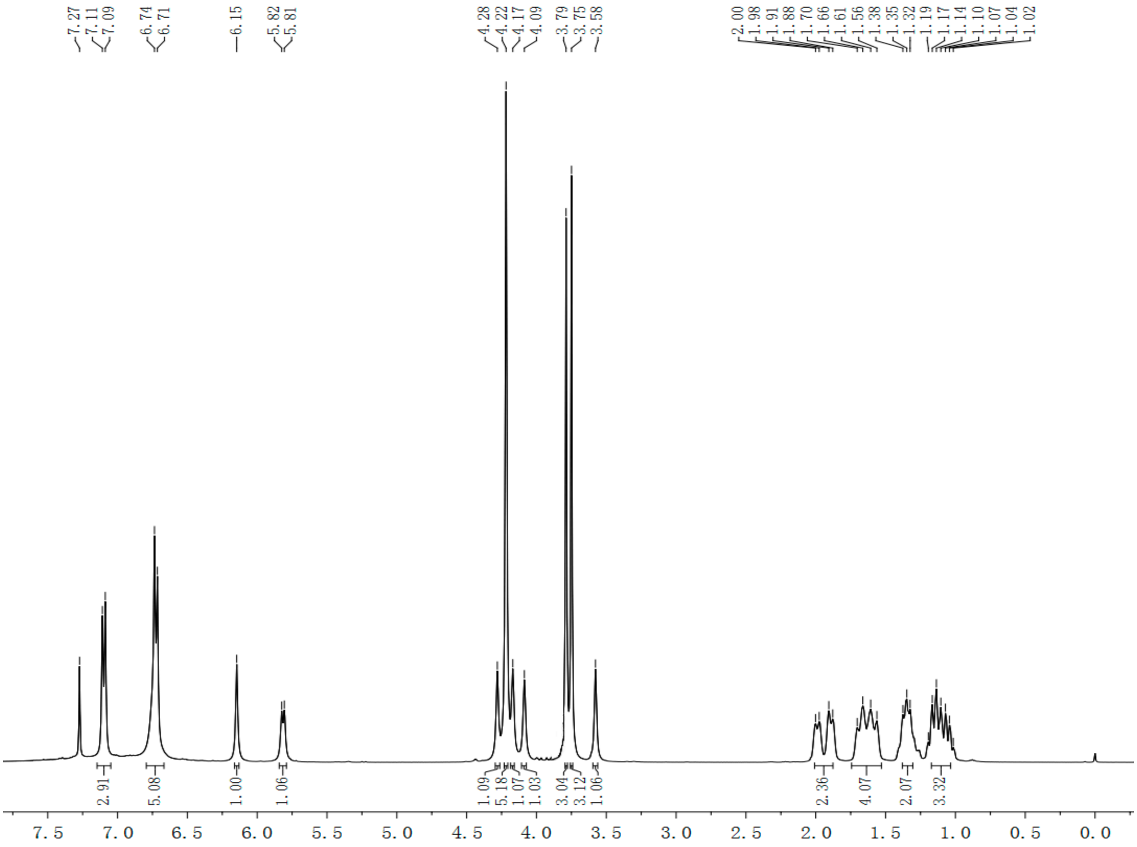

Figure S24. ^{13}C NMR of compound **8**

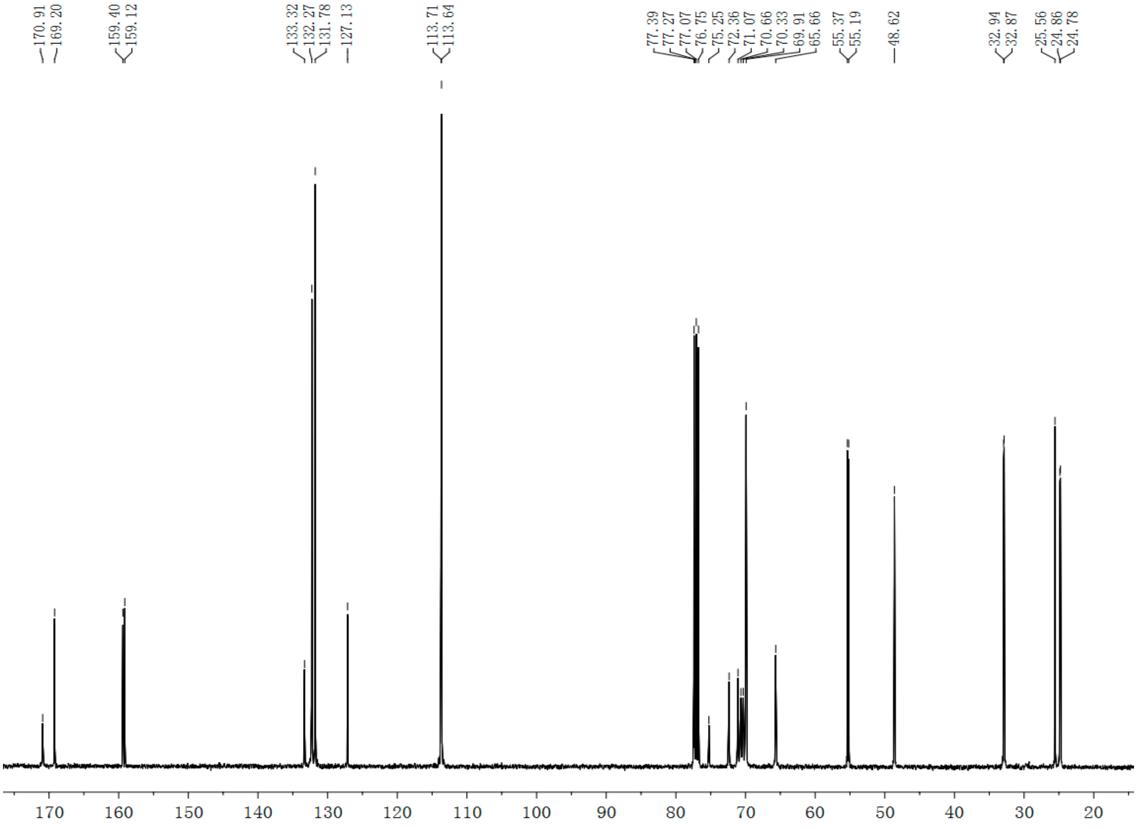

Figure S25. ^{1}H NMR of compound **9**

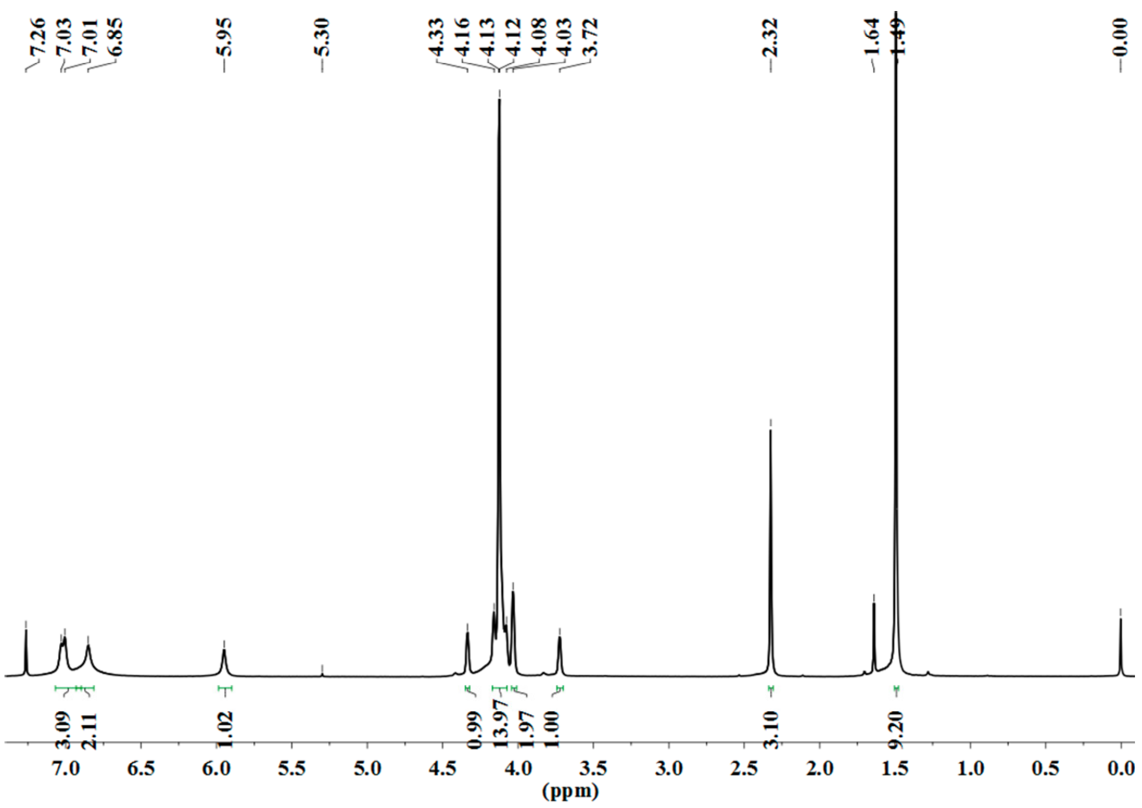

Figure S26. ^{13}C NMR of compound **9**

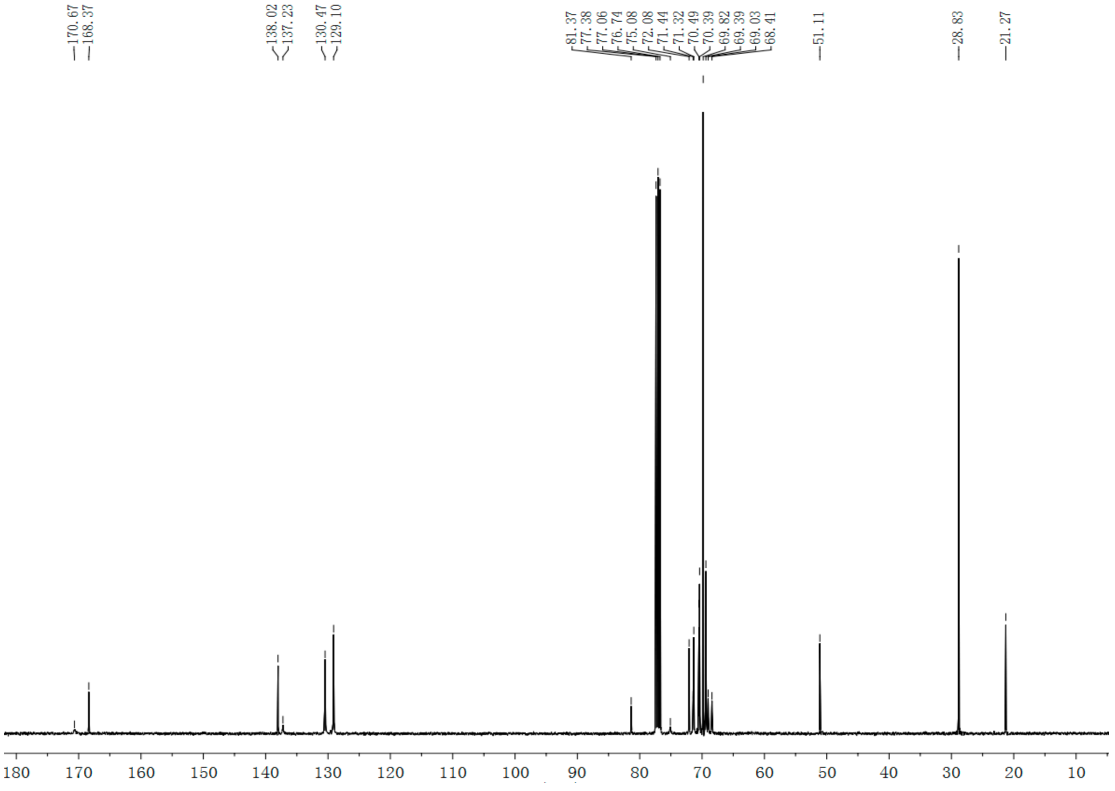

Figure S27. ^{1}H NMR of compound **10**

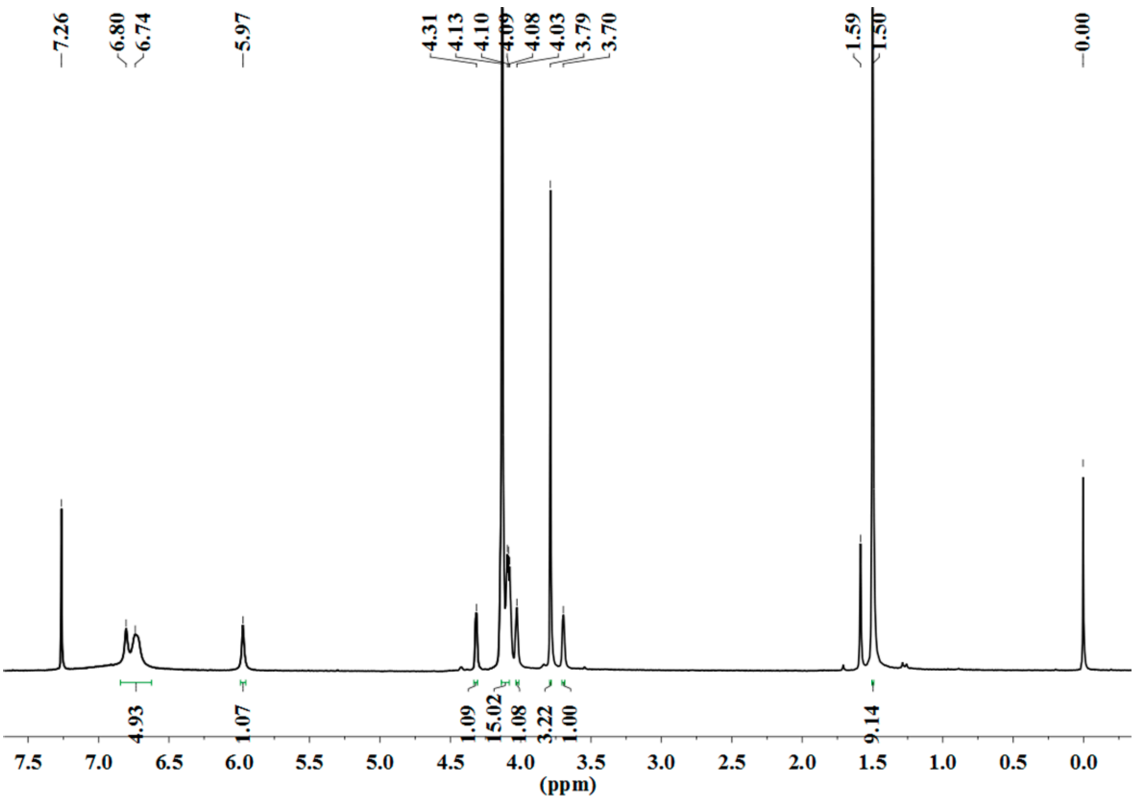

Figure S28. ^{13}C NMR of compound **10**

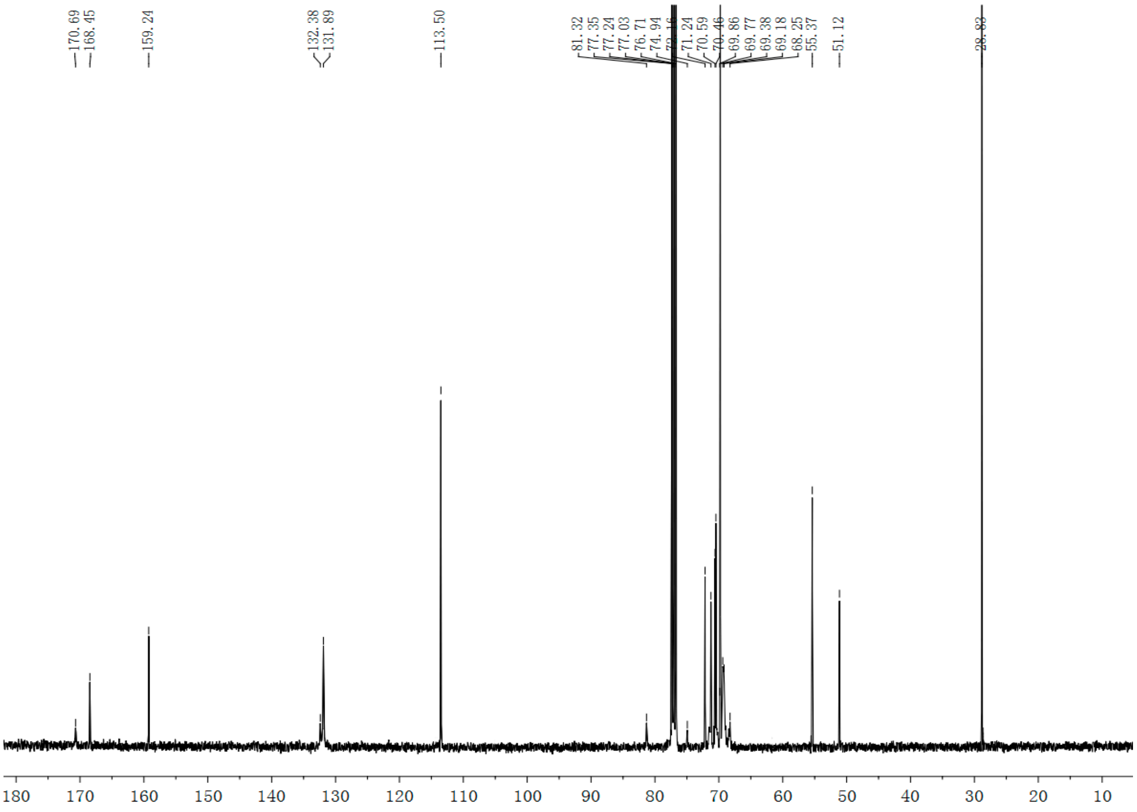

Supplement: Supplementary file 1 [file molecules-22-00737-s001.pdf]
